# Carrier‐Free, Amorphous Verteporfin Nanodrug for Enhanced Photodynamic Cancer Therapy and Brain Drug Delivery

**DOI:** 10.1002/advs.202302872

**Published:** 2024-03-06

**Authors:** John A. Quinlan, Collin T. Inglut, Payal Srivastava, Idrisa Rahman, Jillian Stabile, Brandon Gaitan, Carla Arnau Del Valle, Kaylin Baumiller, Anandita Gaur, Wen‐An Chiou, Baktiar Karim, Nina Connolly, Robert W. Robey, Graeme F. Woodworth, Michael M. Gottesman, Huang‐Chiao Huang

**Affiliations:** ^1^ Fischell Department of Bioengineering University of Maryland College Park MD 20742 USA; ^2^ Laboratory of Cell Biology Center for Cancer Research National Cancer Institute National Institutes of Health Bethesda MD 20892 USA; ^3^ Advanced Imaging and Microscopy Laboratory Maryland Nano Center University of Maryland College Park MD 20742 USA; ^4^ Molecular Histopathology Laboratory Leidos Biomedical Research, Inc. Frederick National Laboratory for Cancer Research Frederick MD 21701 USA; ^5^ Marlene and Stewart Greenebaum Comprehensive Cancer Center University of Maryland School of Medicine Baltimore MD 21201 USA; ^6^ Department of Neurosurgery University of Maryland School of Medicine Baltimore MD 21201 USA

**Keywords:** amorphous drug nanoparticles, blood‐brain barrier, cancer, photodynamic therapy, photosensitizers

## Abstract

Glioblastoma (GBM) is hard to treat due to cellular invasion into functioning brain tissues, limited drug delivery, and evolved treatment resistance. Recurrence is nearly universal even after surgery, chemotherapy, and radiation. Photodynamic therapy (PDT) involves photosensitizer administration followed by light activation to generate reactive oxygen species at tumor sites, thereby killing cells or inducing biological changes. PDT can ablate unresectable GBM and sensitize tumors to chemotherapy. Verteporfin (VP) is a promising photosensitizer that relies on liposomal carriers for clinical use. While lipids increase VP's solubility, they also reduce intracellular photosensitizer accumulation. Here, a pure‐drug nanoformulation of VP, termed “NanoVP”, eliminating the need for lipids, excipients, or stabilizers is reported. NanoVP has a tunable size (65–150 nm) and 1500‐fold higher photosensitizer loading capacity than liposomal VP. NanoVP shows a 2‐fold increase in photosensitizer uptake and superior PDT efficacy in GBM cells compared to liposomal VP. In mouse models, NanoVP‐PDT improved tumor control and extended animal survival, outperforming liposomal VP and 5‐aminolevulinic acid (5‐ALA). Moreover, low‐dose NanoVP‐PDT can safely open the blood‐brain barrier, increasing drug accumulation in rat brains by 5.5‐fold compared to 5‐ALA. NanoVP is a new photosensitizer formulation that has the potential to facilitate PDT for the treatment of GBM.

## Graphical abstract

Photodynamic therapy (PDT) is a promising approach to treating glioblastoma and other cancers. Here, a new pure‐drug nanoparticle of verteporfin (NanoVP) is introduced to significantly improve photosensitizer delivery, enhance PDT efficacy, and increase the permeability of the blood‐brain barrier. This new photosensitizer nanoformulation opens up new possibilities for treating cancer and other challenging lesions protected by the blood‐brain barrier.

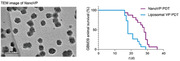

## 1 Introduction

Glioblastoma (GBM) accounts for ≈50% of malignant primary brain tumors and has a five‐year survival rate of only 6.9%.^[^ ^1^ ^]^ Surgical resection improves survival, but ultimately tumor cells that have invaded the surrounding brain tissue limit complete tumor removal without significant neurological injury. These residual GBM cells, usually situated within the 2–3 centimeter margin of the resection cavity,^[^ ^2^ ^]^ lead to nearly universal tumor recurrence and patient death. The current standard of care for patients with GBM consists of surgery followed by radiation and chemotherapy using temozolomide^[^ ^3^ ^]^ and/or implantation of drug‐loaded interstitial wafers (Gliadel) into the resection cavity.^[^ ^4^ ^]^ Even with these aggressive therapies, the median survival for patients with GBM has only marginally improved from 14 to 18 months over the last 20 years.^[^ ^3^, ^5^ ^]^ Considering limited progress in the clinical management of GBM, there is a clear need for novel therapeutic strategies that can enhance patient survival by safely and effectively treating GBM.

Photodynamic therapy (PDT) is a photochemical modality that activates systemically administered photosensitizers, or light‐activated drugs, with temporal and site specificity using light of a specific wavelength. Light‐mediated activation of photosensitizers generates reactive oxygen species (ROS) that induce cell death or targeted biological changes. The most notable use of photosensitizers in GBM is the 2017 FDA approval of fluorescence‐guided surgery with 5‐aminolevulinic acid (5‐ALA)‐induced protoporphyrin IX (PpIX), which resulted in an 80% increase in complete resection of contrast‐enhancing tumor over conventional resection^[^ ^6^ ^]^ and reduced the need for repeat surgery.^[^ ^7^ ^]^ The INDYGO trial of 5‐ALA‐induced PpIX PDT (NCT03048240) demonstrated the feasibility of PDT during GBM resection surgery, but efficacy may ultimately be limited by poor light penetration and PpIX activation beyond the margin of resection.^[^ ^8^ ^]^ Recently, we showed that PDT with liposomal verteporfin (VP) induces PDT effects up to 2 centimeters from the light source in rat brains, while PDT with PpIX only induces PDT effects within 1 centimeter of the light source.^[^ ^9^ ^]^ VP is a photosensitizer that has been used in the clinic for over 2 decades^[^ ^10^, ^11^ ^]^ and is currently in a Phase I/II trial for the treatment of recurrent GBM in a light‐independent manner (NCT04590664). While VP has desirable traits as a photosensitizer for treatment of GBM, this photosensitizer is highly hydrophobic^[^ ^12^ ^]^ and, to date, has required lipid carriers to make clinical delivery feasible.^[^ ^13^, ^14^, ^15^ ^]^

Although liposomes have improved the solubility of VP,^[^ ^16^ ^]^ several studies have shown that liposomal delivery of VP decreases intracellular accumulation within cancer cells by 1–2 orders of magnitude compared to free‐form VP.^[^ ^17^, ^18^, ^19^ ^]^ Liposomes delivered systemically may also be recognized by both the adaptive and innate immune systems. This recognition can result in challenges such as subsequent accumulation and toxicity in the mononuclear phagocyte system, as well as rapid clearance from circulation, among other challenges.^[^ ^20^ ^]^ Free‐form VP is not used clinically because large VP agglomerations created in aqueous buffers negatively impact its pharmacokinetics and singlet oxygen yield.^[^ ^21^ ^]^ Considering the clinical value of liposomes but understanding their limitations for photosensitizer delivery, we sought to develop a lipid‐free nanodrug with efficient VP delivery and release for improved PDT efficacy. Excipient‐free nanodrugs offer the opportunity to deliver higher levels of hydrophobic photosensitizers. They allow for a theoretical drug‐loading capacity of 100% and eliminate the reliance on lipids, polymers, and co‐solvents for drug delivery.

Here, we report an excipient‐free, amorphous nanodrug of VP (termed “NanoVP”), which is a superior PDT agent for the treatment of GBM when compared to liposomal VP and demonstrates enhanced efficacy in blood‐brain barrier (BBB) opening compared to 5‐ALA. The size of NanoVP can be precisely tuned by adjusting the solvent‐to‐antisolvent ratio or changing the initial drug concentration in solvent. NanoVP shows excellent stability over a one‐year storage period in both water and saline solutions. It undergoes dissociation in the presence of biomolecules such as proteins or lipids. Additionally, NanoVP enhances intracellular photosensitizer accumulation, resulting in superior singlet oxygen generation in cells compared to liposomal VP formulations. Ultimately, NanoVP‐PDT outperforms liposomal VP‐PDT in an orthotopic patient‐derived xenograft (PDX) mouse model of GBM. We envision that this novel photosensitizer formulation will advance the role of PDT in the clinical management of GBM and other challenging lesions protected by the BBB.

## 2 Results

### 2.1 Excipient‐Free NanoVP is Amorphous, Monodispersed, and Stable

**Figure 1:**
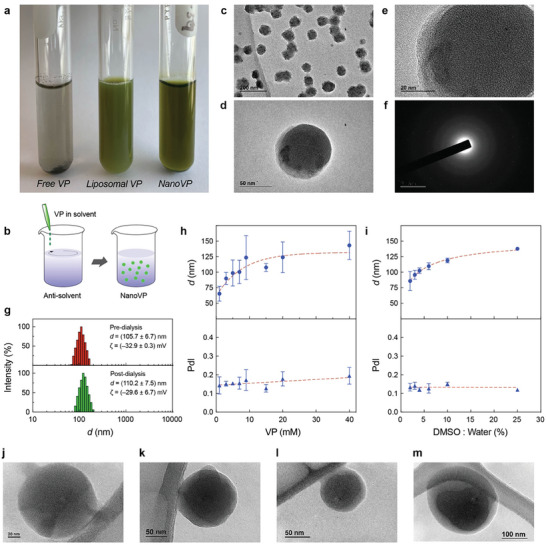
Physical assessment of the amorphous nanodrug of Verteporfin (NanoVP). a) Representative digital image of free‐form VP, liposomal VP, and NanoVP in phosphate‐buffered saline (PBS). b) Schematic depiction of the solvent‐antisolvent precipitation method for preparation of NanoVP. c) Representative transmission electron microscopy (TEM) micrographs (Scale bar = 200 nm) and d,e) close‐up views of monodispersed NanoVP. f) Representative image of selected area electron diffraction of the amorphous NanoVP. g) Representative intensity plots, the average hydrodynamic diameter, and the average zeta potential of NanoVP diameter pre‐dialysis and post‐dialysis. The hydrodynamic diameter and polydispersity index (PdI) of NanoVP h) as a function of initial VP concentration in DMSO or i) as a function of DMSO:water ratio. Representative TEM images of NanoVP formulated as j) 1 mM VP in DMSO with 1:50 DMSO:water ratio, k) 20 mM VP in DMSO with 1:50 DMSO:water ratio, l) 7 mM VP in DMSO with 3:50 DMSO:water ratio, and m) 7 mM VP in DMSO with 1:4 DMSO:water ratio. N ≥ 3. Error bar shows the standard error of the mean.

VP is poorly soluble in water and prone to forming J‐type aggregates in physiologically relevant buffers (**Figure** **1**a).^[^ ^12^ ^]^ To date, intravenous (IV) delivery of VP in the clinic relies on the use of lipids, which serves as a solubilizer. Here, we successfully prepared a well‐dispersed, carrier‐free nanodrug of VP (NanoVP) using the solvent‐antisolvent precipitation technique (Figure 1b). After dissolving VP in 100% dimethylsulfoxide (DMSO) at 7 mM, 1 part sample was added dropwise to 50 parts of stirring water at room temperature, then dialyzed against phosphate‐buffered saline (PBS) at 4 °C. Transmission electron microscopy (TEM) micrographs revealed that NanoVP nanoparticles are spherical‐like, amorphous, monodispersed, and ≈100 nm in diameter (Figure 1c–e; Figure S1, Supporting Information). The lack of electron diffraction further verified that NanoVP is amorphous (Figure 1f). A TEM study using ionic liquid‐treated samples showed that sample dehydration, required for conventional TEM, has a negligible influence on the structure and size of NanoVP (Figure S2, Supporting Information). Alternative synthesis methods, including adding VP in DMSO dropwise into PBS, resulted in uncontrollable agglomeration (Figure S3, Supporting Information).

Dynamic light scattering (DLS) and laser Doppler electrophoresis (LDE) measurements showed that NanoVP formulated under standard conditions (7 mM VP in DMSO; 2% DMSO:water ratio) has a hydrodynamic diameter of 105.7 ± 6.7 nm, a polydispersity index (PdI) of 0.13 ± 0.1, and a zeta potential of −32.9 ± 0.3 mV (Figure 1g). Dialysis for purification and buffer exchange had no significant impact on NanoVP size (110.2 ± 7.5 nm), PdI (0.13 ± 0.2), or zeta potential (−29.6 ± 6.7 mV) (Figure 1g). NanoVP has an entrapment efficiency of 91.6 ± 7.5%, reflecting minimal loss during sample preparation and dialysis. The loading capacity of NanoVP is 706 000 ± 38 000 VP molecules per nanoparticle, which is ≈1455‐fold greater than liposomal VP (485 ± 75 VP molecules per nanoparticle). The size of the NanoVP is tunable between 65 nm and 150 nm by increasing the photosensitizer concentration in solvent (1–40 mM VP in DMSO, Figure 1h) or the DMSO:water ratio (2%–25%, Figure 1i). With these parameters altered, the nanostructure visualized via TEM remained amorphous and spherical (1 mM VP in DMSO with 2% DMSO:water ratio, Figure 1j; 20 mM VP in DMSO with 2% DMSO:water ratio, Figure 1k; 7 mM VP in DMSO with 6% DMSO:water ratio, Figure 1l; 7 mM VP in DMSO with 25% DMSO:water ratio, Figure 1m). However, a starting VP concentration beyond 15 mM or a DMSO:water ratio higher than 6% resulted in multi‐peak size distribution (Figure S4a,b, Supporting Information). NanoVP, synthesized using 1–15 mM VP in DMSO and 2%–20% DMSO:water ratio, was found stable for over one year in PBS (post‐dialysis) and VP in DMSO concentrations from 1–9 mM VP in DMSO were stable for over one year in water (pre‐dialysis) (Figure S5, Supporting Information). We found that NanoVP was unstable after freezing at −20 or −80 °C (Figure S6, Supporting Information). Further studies indicated that NanoVP stability (Supplementary methods) is maintained via electrostatic repulsion forces between the nanoparticles (Figure S7, Supporting Information).

### 2.2 NanoVP is Quenched in Saline and Partially Unquenched in Serum‐Containing Media

**Figure 2:**
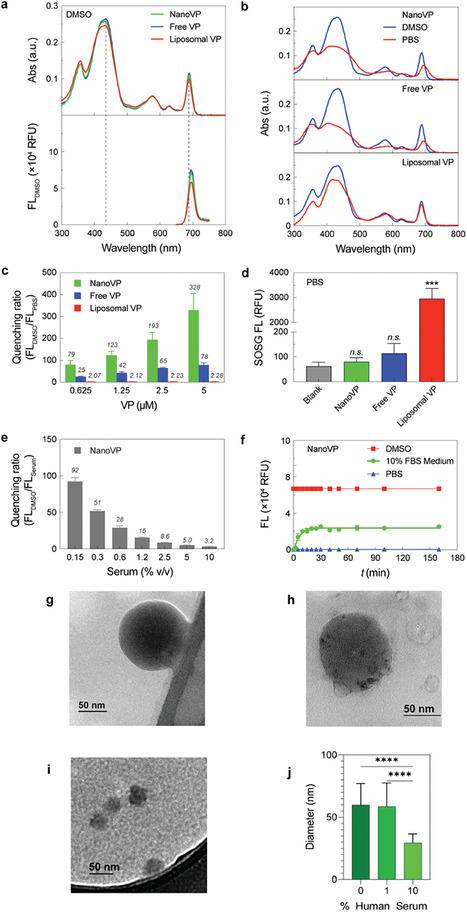
Photochemical characterization of NanoVP, free‐form VP, and liposomal VP. a) Representative absorbance and fluorescence (FL) spectra of 5 µM NanoVP, free‐form VP, and liposomal VP in DMSO. b) Representative absorbance spectra of NanoVP, free‐form VP, and liposomal VP in PBS (red) and DMSO (blue). c) Quenching ratio of NanoVP, free‐form VP, and liposomal VP between 0.625–5 µM. Quenching ratio is defined as the ratio of unquenched fluorescence intensity in DMSO and quenched fluorescence intensity in PBS (FL_DMSO_/FL_PBS_). d) Singlet oxygen production from NanoVP, free‐form VP, and liposomal VP‐mediated PDT (10 J cm^−2^, 10 mW cm^−2^). e) Quenching ratio of NanoVP as a function of serum concentration in PBS. f) The time‐dependent de‐quenching (fluorescence signal recovery; Excitation/Emission: 435/700 nm) of 5 µM NanoVP in PBS, DMSO, and complete cell culture medium supplemented with 10% fetal bovine serum at 37 °C. g) TEM images of NanoVP in 0% human serum, h) 1% human serum, and i) 10% human serum. j) The size of NanoVP in 0%–10% human serum as quantified from TEM images using ImageJ. One‐way ANOVA with multiple comparison test was used to calculate significant differences, where * *P* < 0.05, ** *P* < 0.01, *** *P* < 0.001. N ≥ 3. Error bar shows the standard error of the mean.

VP is a modified porphyrin derivative that displays a chlorin‐type spectrum in organic solvents. In DMSO, the absorption spectrum of VP is characterized by a distinct Q band in the near‐infrared region at 687 nm and a strong Soret band at 435 nm (**Figure** **2**a). The absorption spectra of NanoVP, free‐form VP, and liposomal VP were identical in DMSO, where VP is mainly in its monomeric form. At an excitation wavelength of 435 nm, the fluorescence emission spectra of NanoVP in DMSO can be recorded at ≈700 nm (Figure 2a). In PBS, liposomes maintain some monomeric VP, showing a minimal decrease in the Soret band without a red‐shift displacement of the Q band (Figure 2b). In contrast, when NanoVP is well‐dispersed in PBS, a broadening of the Soret band and a red‐shifted Q band were observed (Figure 2b), suggesting that monodispersed NanoVP consists of self‐assembled J‐aggregates. NanoVP is highly quenched in PBS with a quenching ratio, defined as the ratio of unquenched fluorescence intensity in DMSO and quenched fluorescence intensity in PBS (FL_DMSO_/FL_PBS_), of 328 at 5 µM VP (Figure 2c). This highly quenched NanoVP is likely driven by the spatial confinement of a large number of VP molecules (≈700 000) in a 100 nm diameter nanoparticle. In contrast, the quenching ratios of free‐form VP and liposomal VP are only up to 78 and 2, respectively (Figure 2c). VP quenching can decrease the photochemical production of singlet oxygen (^1^O_2_). Upon light activation (690 nm, 10 J cm^−2^, 10 mW cm^−2^), highly quenched NanoVP and free‐form VP in PBS did not produce any significant amount of ^1^O_2_, as indicated by the minimal singlet oxygen sensor green (SOSG) signal (Figure 2d). On the contrary, light activation of liposomal VP generates an up to 34‐fold higher SOSG signal compared to NanoVP. We also confirmed that light activation of NanoVP, free‐form VP, or liposomal VP results in limited photothermal effects (ΔT = 2–3 °C; Figure S8, Supporting Information).

While NanoVP is quenched and non‐photoactivable in PBS during storage, we hypothesized that NanoVP could be unquenched and photoactivated in serum‐containing media. Figure 2e shows that increasing the serum protein level from 0.15% to 10% v/v decreases the NanoVP quenching ratio in serum from 92 to 3.2. Disaggregation of NanoVP in fetal bovine serum (FBS)‐containing media is a time‐dependent process. After adding NanoVP to 10% v/v FBS‐containing media, a rapid increase in VP fluorescence within 30 minutes is followed by a plateau of the signal, whereupon the signal remains constant at 38% of that fully dissolved in DMSO (Figure 2f). Through TEM, we probed interactions between NanoVP and human serum. Compared to PBS (0% human serum) (Figure 2g), the size of NanoVP as measured from TEM images using ImageJ remains intact after 2 h of 1% human serum incubation (Figure 2h), and a protein corona may form around the NanoVP (Figure 2h). In contrast, NanoVP in 10% human serum has a 49% decrease in diameter in the same time period, but evidence of particles is still visible via TEM (Figure 2i,j).

### 2.3 NanoVP is Unquenched in Cancer Cells, Improving Photosensitizer Uptake and PDT Efficacy

**Figure 3:**
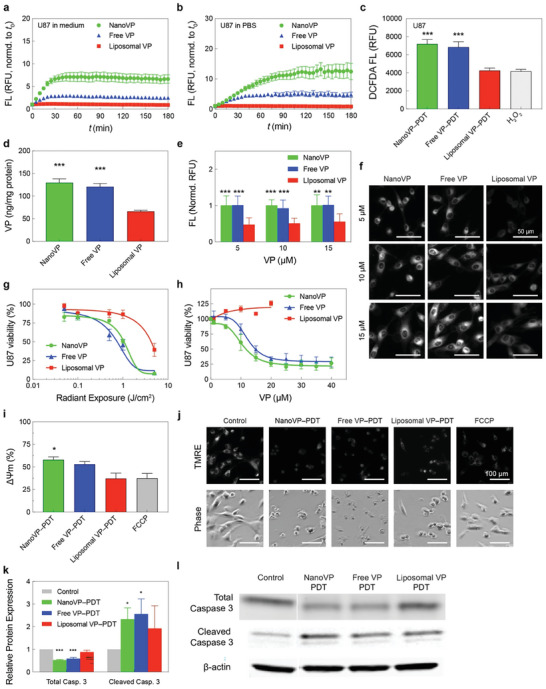
In vitro PDT efficacy of NanoVP in glioblastoma cells. Unquenching of NanoVP by U87 cells in a) culture media and b) in PBS in a time‐dependent manner. c) Light activation (690 nm, 10 J cm^−2^, 50 mW cm^−2^) of NanoVP, free‐form VP, and liposomal VP resulted in reactive oxygen species (ROS) production in U87 cells. d) The VP concentrations in U87 cells were determined via extraction method at 24 h post‐incubation with 0.25 µM NanoVP, free‐form VP, or liposomal VP. e) Quantitative analyses of NanoVP, free‐form VP, and liposomal VP fluorescence signals in U87 cells. Fluorescence signals were normalized to the largest signal for each concentration. f) Representative fluorescence images of NanoVP, free‐form VP, and liposomal VP accumulated within U87 cells. Scale bar = 50 µm. g) U87 cell viability measured via MTT assay at 24 h after PDT using NanoVP, free‐form VP, or liposomal VP (690 nm, 0–5 J cm^−2^, 10 mW cm^−2^). h) Dark toxicity of NanoVP, free‐form VP, and liposomal VP in U87 cells. Cell viability was measured via MTT assay 72 h after photosensitizer incubation. j) Representative fluorescence images of the TMRE probe and phase constant images of U87 cells at 30 min after PDT (690 nm, 10 J cm^−2^, 50 mW cm^−2^). i) Mitochondrial membrane potential depolarization was quantified via TMRE probe 1 h after PDT (10 J cm^−2^, 50 mW cm^−2^) using NanoVP, free‐form VP, or liposomal VP. Mitochondrial membrane potential depolarization was calculated using the formula: ΔΨm = 1‐(Tf/Tc), where Tf is the TMRE fluoresce signal from FCCP or a treatment group, and Tc is the TMRE fluoresce signal from the control group. k) Quantification of total and cleaved caspase 3 expressions in U87 cells at 1 h post‐PDT (690 nm, 10 J cm^−2^, 50 mW cm^2^). N ≥ 3. A two‐tail (total) and one‐tail (cleaved) t‐test was used to calculate significant differences. l) Representative immunoblotting showed changes in total and cleaved caspase 3 expressions in U87 cells at 1 h after PDT. One‐way ANOVA with multiple comparison tests was used to calculate significant differences, where * *P* < 0.05, ** *P* < 0.01, and *** *P* < 0.001. N ≥ 3. Error bar shows the standard error of the mean.

We sought to understand the unquenching and photochemical activity of NanoVP in cancer cells. U87 cells both in medium (**Figure** **3**a) and in PBS (Figure 3b) were able to unquench NanoVP in a time‐dependent manner, with superior fluorescence recovery compared to free‐form VP and liposomal VP. To check the recovery of photochemical activity of NanoVP in U87 cancer cells, dichlorodihydrofluorescein diacetate (DCFDA) was used as a fluorescent probe for the detection of intracellular ROS generation (Figure 3c). Surprisingly, upon light activation (690 nm, 10 J cm^−2^, 50 mW cm^−2^), NanoVP produced significantly higher intracellular ROS (≈2‐fold), compared to that of liposomal VP and the positive control (100 µM H_2_O_2_), despite identical treatment conditions.

To investigate why NanoVP‐PDT treatment resulted in greater intracellular ROS generation, we first probed intracellular photosensitizer concentration. The therapeutic efficacy of PDT depends on the concentration of photosensitizer in cancer cells. U87 cells treated with NanoVP had twice as much intracellular VP (129.8 ± 29.1 ng mg^−1^ of protein) compared to liposomal VP (66.2 ± 8.1 ng mg^−1^ of protein) (Figure 3d). We further confirmed enhanced uptake of NanoVP via fluorescence imaging of U87 cells treated with free VP, NanoVP, and liposomal VP, finding enhanced uptake of free VP and NanoVP over liposomal VP at all concentrations (Figure 3e,f). We found a 49.9% increase in uptake of NanoVP in noncancerous 3T3 mouse fibroblast cells compared to cells treated with liposomal VP (Figure S9a, Supporting Information). Intracellular ROS generation and cellular uptake of NanoVP was similar to that observed from free VP, which is clinically nonviable due to the organic solvent carrier that is needed to solubilize VP.

Increased uptake of NanoVP and increased singlet oxygen production results in a 400% stronger anti‐GBM PDT effect than liposomal VP, as evaluated by MTT (Figure 3g). NanoVP and free‐form VP had a half‐maximal inhibition concentration (IC_50_) of 0.2 µM × J cm^−2^ in U87 cells, while liposomal VP had a 4‐fold higher IC_50_ of 0.8 µM × J cm^−2^. PDT IC_50_ in noncancerous 3T3 cells was 0.4 µM × J cm^−2^ for NanoVP and 0.8 µM × J/cm^2^ for liposomal VP (Figure S9b, Supporting Information). These IC_50_ values indicate a potentially greater therapeutic index for PDT using NanoVP (0.2 µM × J cm^−2^ difference in IC_50_s between cancerous and noncancerous cells) compared to PDT using liposomal VP (no differences in IC_50_s). Considering recent efforts using VP in a light‐independent manner to treat glioblastoma (NCT04590664),^[^ ^22^, ^23^, ^24^ ^]^ we treated U87 and 3T3 cells with higher concentrations of NanoVP in complete darkness. NanoVP exhibited an IC_50_ of 10.5 µM in U87 cells and 3T3 cells; free VP performed similarly (Figure 3h; Figure S9c, Supporting Information). In contrast, liposomal VP exhibited no dark cytotoxicity in the range of concentrations tested, presumably due to the poor intracellular VP accumulation.

We next explored the mechanisms of NanoVP‐mediated PDT cell killing. VP preferentially accumulates in mitochondria and light activation of VP depolarizes the mitochondrial membrane potential (ΔΨm) to trigger apoptosis.^[^ ^25^, ^26^, ^27^ ^]^ By tetramethylrhodamine ethyl ester (TMRE) assay, NanoVP‐PDT (690 nm, 10 J cm^−2^, 50 mW/cm^−2^) induced the largest degree of ΔΨm depolarization (Figure 3i,j). P‐triflouromethoxyphenylhydrazone (FCCP), a mitochondrial oxidative phosphorylation uncoupler, was used as a positive control. Relative to untreated controls, NanoVP‐PDT depolarizes ΔΨm by nearly 60%, while liposomal VP‐mediated PDT results in depolarization of ΔΨm by ≈35%. PDT‐induced mitochondria damage triggers the intrinsic apoptosis pathway, leading to caspase‐3 activation.^[^ ^28^, ^29^, ^30^, ^31^ ^]^ Immunoblotting results indicate that PDT using NanoVP or free‐form VP induced a ≈2.4‐fold increase in cleaved caspase‐3 one‐hour post‐light activation (Figure 3k,l). In addition, NanoVP and free‐form VP‐mediated PDT significantly reduced total (pro) caspase‐3 levels by ≈50%, resulting in a cleaved‐to‐total caspase‐3 ratio of ≈4.4. Liposomal VP‐PDT resulted in the lowest ratio of ≈2.2. Next, we investigated the interaction of NanoVP with other cellular components. Neutral red uptake (NRU) assay revealed that light‐activation (690 nm, 1 J cm^−2^, 50 mW cm^−2^) of NanoVP, free‐form VP, or liposomal VP does not affect lysosomal integrity in U87 cancer cells (Figure S10, Supporting Information). Previously, we have shown that VP can be effluxed by ATP‐binding cassette (ABC) drug transporters (breast cancer resistance protein, ABCG2; and P‐glycoprotein, P‐gp), reducing PDT efficacy.^[^ ^18^ ^]^ Using a human breast cancer cell line MCF‐7, and its sub‐lines ABCG2‐overexpressing MCF‐7 MX100 and or P‐gp‐overexpressing MCF‐7 TX400,^[^ ^18^, ^32^ ^]^ we found that NanoVP remains a substrate of ABCG2 and P‐gp (Figure S11, Supporting Information).

### 2.4 NanoVP‐PDT Enhances Acute Tumor Control in a Xenograft Mouse Model of GBM

**Figure 4:**
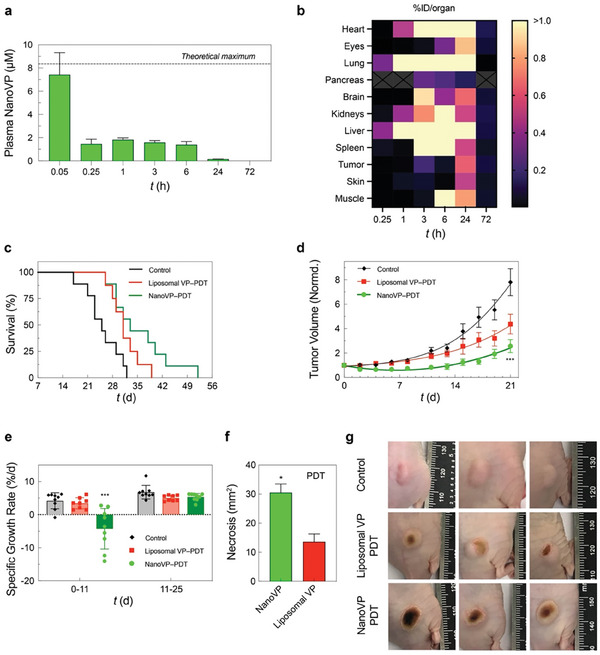
The phototoxicity and biodistribution of NanoVP in U87 glioblastoma xenograft mouse model. a) Pharmacokinetics of NanoVP after 0.5 mg kg^−1^ IV administration. b) NanoVP biodistribution as measured via HPLC‐MS/MS at various time points after 0.5 mg kg^−1^ IV NanoVP administration to mice bearing flank GBM39 tumors, expressed as percent injected dose per organ. Grey boxes with an “X” indicate no data. c) Kaplan‐Meier plot of tumor diameter greater than 1.5 cm (N = 7–8 animals per group). PDT treatment (100 J cm^−2^, 100 mW cm^−2^) was initiated ≈14 days after subcutaneous U87 cancer cell implantation when tumor volumes reached ≈100 mm^3^. Mice were randomized into groups that received i) no‐treatment, ii) Liposomal VP (0.5 mg kg^−1^), and iii) NanoVP (0.5 mg kg^−1^). d) Tumor volume was longitudinally monitored and calculated using the standard estimation formula, V = ½ × length × width^2^, where length equals the maximum tumor diameter in millimeters and width equals the diameter that is perpendicular to the length. Tumor volumes were normalized to the initial volume at the time of treatment. e) The specific growth rate (SGR) of tumors 0–11 days and 11–25 days post‐PDT were determined using the following formula: SGR = (1/V)(dV/dt), where V is tumor volume and t is time. f) Quantification of the dark, necrotic surface area above the tumor that was impacted by PDT treatment. g) Representative digital images of tumors at 6 days post‐PDT. One‐way ANOVA with multiple comparison test was used to calculate significant differences, where *P < 0.05, **P < 0.01, and ***P < 0.001. N ≥ 3. Error bar shows the standard error of the mean.

NanoVP pharmacokinetics were evaluated in mice. Animals received 0.5 mg kg^−1^ NanoVP via tail vein injection. We observed an initial spike in <5 min, followed by a plateau in plasma levels from 15 min post‐injection to 6 h post‐injection (**Figure** **4**a). Complete clearance was observed at 72 h. The pharmacokinetic profile of NanoVP was found to be similar to that of both VP in DMSO and liposomal VP as reported by others.^[^ ^33^, ^34^ ^]^ Qualitative comparison of biodistribution at 2 and 24 h post‐injection in mice bearing flank U87 tumors indicates that NanoVP and liposomal VP may have similar biodistribution profiles Figure S12, Supporting Information). Quantification of VP levels in mice bearing flank GBM39 tumors receiving 0.5 mg kg^−1^ NanoVP IV revealed persistently high levels in highly vascularized tissues (e.g., heart, lung, kidneys, liver, spleen), and tumor‐to‐normal tissue (skin) ratios of 0.3–1.8 around the time of intended treatment (1–3 h post‐injection) (Figure 4b; Table S1, Supporting Information). Clearance was observed by 72 h, consistent with pharmacokinetic data.

We next assessed the efficacy of NanoVP‐PDT in a U87 flank tumor PDT mouse model. Once tumors reached ≈100 mm^3^, mice received 0.5 mg kg^−1^ NanoVP or liposomal VP via tail vein injection. After a 2 h drug‐light interval, tumors received top‐down illumination for PDT (690 nm, 100 J cm^−2^, 100 mW cm^−2^). In this model, a single cycle of NanoVP‐PDT modestly improved animal survival (median survival: 33 days), compared to no‐treatment control (median survival: 25) and liposomal VP‐PDT (median survival: 31 days) (Figure 4c). From 2–11 days after treatment, NanoVP‐PDT reduced tumor volume by up to 35%, while tumors continued to grow in no‐treatment and liposomal VP‐PDT control groups (Figure 4d). Tumors treated with NanoVP‐PDT had a specific growth rate (SGR) of −4.3%/day from day 0–11 post‐PDT. At 11 days post‐treatment, tumor regrowth began, and the SGR increased to 5.3%/day, similar to the SGR observed in control and liposomal VP‐PDT groups (Figure 4e). In addition to changes in tumor volume, noticeable visual differences on tumor surfaces were observed (Figure 4f). At six days post‐treatment, NanoVP‐PDT resulted in a significantly larger dark, necrotic area that covered most of the tumor (30.5 mm^2^), while liposomal VP‐PDT led to a smaller (13.6 mm^2^) and more localized necrotic patch (Figure 4f,g). Treatment of flank tumors was well tolerated with steady weight gain in all treatment groups (Figure S13, Supporting Information).

### 2.5 NanoVP‐PDT Prolongs Survival in an Orthotopic GBM PDX Mouse Model

**Figure 5:**
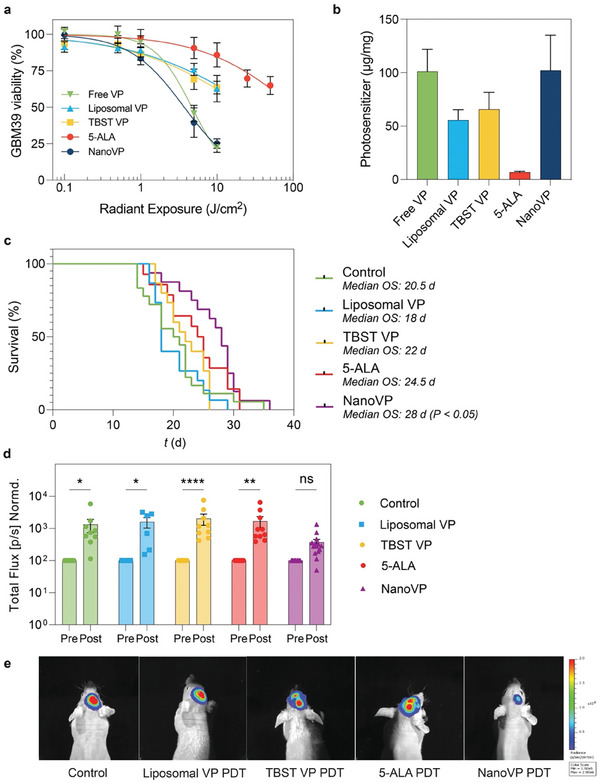
PDT management of orthotopic patient‐derived xenograft glioblastoma. a) In vitro GBM39 cell viability measured via CellTiter Glo® 24 h after PDT treatment (VP formulations: 0.25 µM, 690 nm, 20 mW cm^−2^, 0–10 J cm^−2^; 5‐ALA: 5 mM, 635 nm, 50 mW cm^−2^, 0–50 J cm^2^). b) The uptake of photosensitizer in GBM39 cells in vitro was measured via extraction after 24 h of incubation with 1 µM free VP, liposomal VP, TBST VP, NanoVP, or 5 mM 5‐ALA. In the case of 5‐ALA‐treated cells, PpIX was quantified. For in vivo experiments, 5 × 10^5^ GBM39 cells were implanted intracranially. After two weeks, interstitial PDT with liposomal VP, NanoVP, or TBST VP (0.5 mg kg^−1^ IV, 2 h drug‐light interval, 12 J cm^−1^, 40 mW cm^−1^) or 5‐ALA (20 mg kg^−1^ IV, 4 h drug‐light interval, 60 J cm^−1^, 100 mW cm^−1^) was performed. c) Kaplan‐Meier plot for survival after treatment with PDT with liposomal‐PDT or NanoVP‐PDT; *P* < 0.05 by Log‐rang test. d) GBM39 cells express luciferase; total bioluminescence was used as a proxy for tumor size at 1‐week post‐treatment (day 21) and normalized to pretreatment values (day 14) (N = 8 for Control, N = 6 for liposomes, N = 9 for TBST VP, N = 10 for 5‐ALA, N = 14 for NanoVP). Two‐way ANOVA with multiple comparison test was used to calculate significant differences, where * *P* < 0.05, ** *P* < 0.01, **** *P* < 0.0001. Error bar shows the standard error of the mean. e) Representative images showing better tumor control with NanoVP at day 21, 1‐week post‐treatment.

While U87 is an established GBM model, we further evaluated the efficacy of NanoVP in GBM39 cells, which is a flank‐passaged patient‐derived xenograft (PDX) model.^[^ ^35^ ^]^ We first evaluated the efficacy of NanoVP‐PDT in GBM39 cells cultured in vitro. Based on our pharmacokinetics results and the apparent dissociation of NanoVP in the blood, we hypothesized that NanoVP may functionally be a molecularly dispersed formulation of VP. We identified tris‐buffered tween‐20 (TBST) as a solubilizing aqueous solution for VP and added this molecularly dispersed formulation of the drug as a control (Figure S14, Supporting Information). We also compared NanoVP‐PDT, free VP (in DMSO)‐PDT, TBST VP‐PDT, and liposomal VP‐PDT to 5‐ALA‐PDT, which is currently in clinical trials for GBM (NCT04391062, NCT04469699, NCT03897491). In GBM39 cells, we found that NanoVP‐PDT has the lowest IC_25_ of ≈0.38 µM × J cm^−2^, outperforming free VP‐PDT (IC_25_ ≅ 0.62 µM × J cm^−2^), TBST VP‐PDT (IC_25_ ≅ 0.86 µM × J cm^−2^), liposomal VP‐PDT (IC_25_ ≅ 1 µM × J cm^−2^), and 5‐ALA‐PDT (IC_25_ ≅ 110 mM × J cm^−2^) (**Figure** **5**a). This may be partially attributable to increased photosensitizer uptake with NanoVP over liposomal VP, TBST VP, and 5‐ALA (PpIX) (Figure 5b).

To evaluate the potential of NanoVP to control orthotopic GBM and improve animal survival, we injected 5 × 10^5^ GBM39 PDX cells expressing luciferase into the right striatum of nude mice. We utilized the GBM39 PDX, which is serially passaged in mice rather than cultured in dishes, to better recapitulate the tumor biology observed in patients. Fourteen days post‐injection, when IVIS signal was robust and when mortality was first observed, we performed interstitial PDT (0.5 mg kg^−1^ NanoVP, liposomal VP, or TBST VP via tail vein injection; 12 J cm^−1^, 40 mW cm^−1^, 690 nm, 2 h drug‐light interval) by inserting a laser fiber (MedLight RD10 fiber, Modulight ML6600 laser) through the burr hole initially used to inject cells. Tolerable conditions for VP‐PDT were established via a dose‐escalation study and 5‐ALA‐PDT conditions were established based on current clinical trial parameters. 5‐ALA is dosed at 20 mg kg^−1^ with a 4 h drug‐light interval in the clinic, with ≈10 min added to surgical time and irradiances and fluences exceeding those explored in this study (Table S2, Supporting Information). Body weight (Figure S15, Supporting Information) as part of a body condition score was used to monitor animal health over the course of the study. We observed no increase in temperature at the site of PDT, as measured by a FLIR infrared camera under conditions used to perform VP‐PDT (690 nm, 40 mW cm^−1^), but we did observe an increase in temperature at the site of PDT with 5‐ALA‐PDT (635 nm, 100 mW cm^−1^) treated mice (Figure S16a, Supporting Information). Upon examination of the laser fiber itself, we identified a significant increase in temperature during and immediately after 5‐ALA‐PDT using 635 nm laser (Figure S16b, Supporting Information). Ultimately, we observed a potential zone of necrosis surrounding the laser insertion site in 5‐ALA PDT‐treated mice, while the neuropil recovered after PDT in control and NanoVP‐PDT mice (Figure S16c, Supporting Information).

We observed a 7‐day median survival benefit in mice treated with NanoVP over untreated mice (28 days versus 21 days, *P* < 0.05, Figure 5c) and a 10‐day median survival benefit over mice treated with liposomal VP‐PDT (28 days versus 18 days, *P* < 0.05, Figure 5c). This significant survival benefit was likely due to the superior acute control of tumor growth. When normalized to pre‐treatment bioluminescent signal, the bioluminescent signal one week post‐treatment had the smallest increase in signal in the NanoVP‐PDT group (mean 369% increase), while liposomal VP‐PDT (mean 1590% increase), control (mean 1270% increase), TBST VP (mean 2030% increase), and 5‐ALA PDT (mean 1680% increase) groups had significant increases in the bioluminescent signal (Figure 5d,e). While orthotopic interstitial PDT with NanoVP provided a significant median survival benefit and significant reduction of tumor burden, these results point out challenges in achieving long‐term improvements in treatment response for GBM, highlighting the need for developing combination strategies to provide durable tumoricidal control in the future.

### 2.6 NanoVP‐PDT Safely and Selectively Improves Blood‐Brain Barrier Permeability in a Rat Model

**Figure 6:**
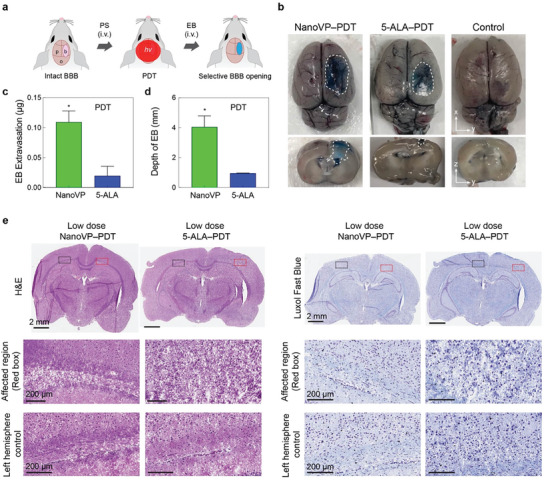
Photodynamic BBB opening within the rat brain. a) Schematic depiction of experimental design. NanoVP (0.25 mg kg^−1^) or 5‐aminolevulinic acid (5‐ALA, 20 mg kg^−1^) were IV administered 30 minutes before PDT (NanoVP: 690 nm, 80 J cm^−2^; 5‐ALA: 635 nm, 80 J cm^−2^; 85 mW cm^−2^) was performed on the exposed right brain hemisphere. After 90 min, Evans blue was IV administered to the rats and circulated for 30 min prior to brain harvesting. b) Representative top and cross‐sectional images of Evans blue within the brain after PDT‐induced BBB opening c) Quantification of Evans blue extracted from the right brain hemispheres (N ≥ 3 rats per group, background subtracted). d) Quantification of maximum depth that extravasated Evans blue can be visualized within the brain. e,f) Representative histopathology of rat brain tissue after traditional and low‐dose PDT. Photomicrographs of brain sections stained with Hematoxylin and eosin (H&E) and Luxol fast blue. Two tail t‐test was used to calculate significant differences, * *P* < 0.05. Error bar shows the standard error of the mean.

The BBB remains a critical obstacle to the effective treatment of GBM and other central nervous system diseases. We previously demonstrated that low‐dose PDT priming allows transient opening of the BBB through reversible modulation of endothelial cell‐cell junction phenotype.^[^ ^36^ ^]^ After demonstrating the superiority of NanoVP‐PDT in our mouse model of GBM, we investigated the ability of low‐dose NanoVP‐PDT to permeabilize the BBB more distantly from the site of light irradiation. In the clinic, this may permit enhanced drug delivery to tumor cells intercalated with healthy brain stroma that are not close enough to the light source to be killed. Here, we evaluated the utility and safety of low‐dose NanoVP‐PDT priming for spatially targeted BBB opening in healthy rats with intact BBB to avoid confounding BBB permeabilization due to surgery or the presence of the tumor (**Figure** **6**a). Because PDT with NanoVP conferred a survival benefit over PDT with liposomal VP, we compared NanoVP‐PDT with 5‐ALA (Gliolan)‐PDT, an FDA‐approved photosensitizer prodrug routinely used for PDT opening of the BBB, which served as benchmark group in our study. Doses for photosensitizer and light used to permeabilize the BBB were informed by previous preclinical vessel permeabilization doses and doses established to be safe in the clinic (Table S3, Supporting Information). We identified a 5.5‐fold increase of total Evan Blue, a non‐BBB penetrant model drug, accumulation in NanoVP‐PDT treated brain compared to 5‐ALA‐PDT treated brain tissues (*P* < 0.05) (Figure 6b,c). The spatiotemporal selectivity of PDT confines Evan Blue delivery to the right‐brain hemisphere where the light is directed, thereby reducing normal tissue damage. The use of the longer activation wavelength, 690 nm, to photoactivate NanoVP for deeper tissue penetration is also an advantage over using 5‐ALA‐induced protoporphyrin IX (PpIX), photoactivatable at 635 nm (Figure 6d). We showed that 690 nm light activation of NanoVP improves Evan Blue delivery at further depths in rat brains (visible up to 4–5 mm), compared to 635 nm light activation of 5‐ALA‐induced PpIX (visible up to 1 mm). Histological changes of brain tissue after PDT were studied microscopically on sections stained with H&E or Luxol Fast blue. Compared to the left‐brain hemisphere that did not receive treatment due to the intact skull, the right‐brain hemisphere did not have any signs of low‐dose PDT priming‐induced damage. H&E staining revealed no detectable lesions at the site of treatment (right hemisphere) and Luxol fast blue staining was symmetrical with no evidence of demyelination (Figure 6e). Alternatively, rats that received traditional high‐dose PDT with either 5‐ALA or NanoVP had signs of brain damage within the neuropil parenchyma (brain cortex) (Figure S17, Supporting Information). This result agrees with previous studies that show dose escalation results in increased signs of edema and brain damage.^[^ ^37^ ^]^

## 3 Discussion

Since receiving FDA approval in 2001, PDT using liposomal VP has benefitted hundreds of thousands of patients globally with wet age‐related macular degeneration.^[^ ^38^ ^]^ Liposomal VP has been tested clinically to treat a wide range of cancers, including primary breast, retinoblastoma, and refractory brain tumors. Liposomal VP is currently being evaluated in patients with unresectable solid pancreatic tumors or advanced pancreatic cancer (NCT03033225), and patients with recurrent high‐grade EGFR‐mutated glioblastoma (NCT04590664) in a light‐independent manner. Pure drug nanoparticle delivery systems offer tools to improve the pharmacokinetic profile of hydrophobic drugs and minimize the reliance on solubilizing agents.^[^ ^39^ ^]^ In this study, we report a new pure‐drug nanoparticle of VP (NanoVP) that significantly improves photosensitizer delivery to cancer cells. Our in vivo findings and results show the safety, feasibility, and potential utility of NanoVP for PDT of gliomas, as well as BBB opening to enhance drug delivery with no evidence of microscopic injury in normal tissues.

Solvent‐antisolvent precipitation is a simple and reproducible formulation strategy to synthesize stable nanoparticles of VP where miscible solvents create local supersaturation of VP, which is thermodynamically unfavorable and leads to amorphous precipitation or crystallization. Altering the concentration of VP in solvent or the DMSO:water ratio permits size tunability between 65 and 150 nm, which is consistent with trends observed with nanodrug paclitaxel.^[^ ^40^ ^]^ Synthesis optimization revealed that monodispersed NanoVP can be produced using an initial VP concentration of less than 15 mM and a DMSO:water ratio below 6%. However, this synthesis is only successful in water, potentially because salts in PBS either served as nucleation sites or changed the charge of the dispersion.^[^ ^41^ ^]^ The nano‐range size and the amorphous structure of NanoVP are strengths for its use in PDT. VP in organic solvent will aggregate uncontrollably when introduced to salt‐containing fluids such as serum. Managing this aggregation to form nanosized particles allows for improved control over pharmacokinetics and dissociation kinetics. The ability of a drug to form nanocrystals or nanoaggregates is a material property. Drugs forming nanocrystals tend to have high saturation solubility and high stability post‐injection due to the minimized Gibbs free energy,^[^ ^41^ ^]^ which has been leveraged for extended release of antiretroviral therapies,^[^ ^42^, ^43^, ^44^ ^]^ anticancer drugs,^[^ ^45^, ^46^ ^]^ and other medications.^[^ ^47^ ^]^ In contrast, amorphous nanodrugs generally possess a higher saturation solubility and, consequently, an increased dissolution velocity (creation of high *C_max_*, reduction of *t_1/2_*) compared to equally sized nanocrystals.^[^ ^48^ ^]^ The expedient dissociation and subsequent retention of photoactivity of NanoVP in the presence of biomolecules overcomes the challenge of self‐quenching that other densely packed porphyrins have faced due to their amorphous structure. The amorphous particles are stable enough to be stored and delivered in aqueous buffers, but permit rapid dissociation, likely allowing VP to redistribute to proteins and lipids.^[^ ^49^ ^]^

Previous studies have shown that liposomes hinder drug uptake^[^ ^19^, ^50^, ^51^ ^]^ compared to free‐form or other carrier‐free nanodrug formulations. For example, free‐form doxorubicin^[^ ^52^ ^]^ and paclitaxel^[^ ^50^ ^]^ are taken up by cells to a greater degree than their liposomal formulations. We speculate that NanoVP had superior uptake compared to liposomal VP because NanoVP is readily dissociated into its molecular form in the presence of the serum components or cells, permitting passive diffusion through the cell membrane,^[^ ^53^, ^54^ ^]^ while liposomes must be endocytosed.^[^ ^55^ ^]^ As a direct result of improved cellular accumulation, NanoVP was found to be a more potent PDT agent than the traditional liposomal VP. Upon internalization, NanoVP‐PDT effectively produces intracellular ROS, induces mitochondrial membrane potential (ΔΨm) depolarization, and initiates intrinsic apoptosis to a greater degree than liposomal VP‐PDT. We observed a wider therapeutic index with NanoVP‐PDT compared to liposomal VP‐PDT, likely attributable to a greater difference in uptake between U87 and 3T3 cells for NanoVP (3‐fold increase) compared to liposomal VP (2‐fold increase). A future direction includes examining the specific uptake mechanisms of NanoVP and liposomal VP in cancerous and noncancerous cells. Typically, PDT requires very low doses of photosensitizers to be effective. Recent clinical and preclinical studies have reported that higher concentrations of VP can induce “dark” cytotoxicity in GBM cells,^[^ ^22^, ^23^, ^24^ ^]^ characterized by downregulation of Bcl‐2, disruption of the YAP/TAZ‐TEAD complex interaction, and induction of cancer cell death without light activation.^[^ ^22^, ^23^ ^]^ We demonstrated that the enhanced cellular uptake of NanoVP resulted in superior non‐PDT (“dark”) killing effects, as liposomal VP alone did not inhibit cancer growth at the same concentrations, likely due to poor intracellular uptake.

In both subcutaneous and orthotopic GBM mouse models, NanoVP‐PDT effectively reduced initial tumor growth and prolonged animal survival, outperforming liposomal VP‐PDT. Although VP‐PDT has been used successfully in the clinic to treat various cancers,^[^ ^56^ ^]^ a single cycle of PDT, like most cancer regimens, is insufficient to achieve a long‐term cure. In our study, NanoVP increased the median survival time of mice bearing intracranial GBM tumors by over a week (55%). This result is similar to our previous study, where we demonstrated a single dose of VP‐PDT (0.25 mg kg^−1^) could reduce OVCAR‐5 tumor volume by up to 55% but did not cure the tumor.^[^ ^57^ ^]^ Biodistribution in the flank tumor model indicated moderate intratumoral accumulation, as anticipated with a non‐targeted nanoparticle. Interestingly, the brain‐to‐skin ratios around the intended time to treat (1–3 h post‐injection) were 0.1–8.4, indicating potentially high bioavailability in the brain. Future work will further evaluate biodistribution in the brain, particularly considering the potential to enhance brain delivery via PDT‐mediated BBB permeabilization. In our study, we included 5‐ALA‐PDT, which is currently under investigation in the clinic, to benchmark NanoVP‐PDT against a clinically relevant PDT approach. Improved efficacy of NanoVP‐PDT over 5‐ALA‐PDT may be attributable to greater intracellular accumulation of the photosensitizer, as observed in vitro. We also compared NanoVP to TBST VP, a molecularly dispersed formulation that was identified as an appropriate control. The improved performance of NanoVP‐PDT over TBST VP‐PDT may be attributable to changes in the association of VP with plasma molecules, resulting in differing biodistribution and ultimately differing efficacy of VP‐PDT, as has been observed historically with liposomal VP.^[^ ^33^, ^49^, ^58^, ^59^ ^]^ Future work will investigate the behavior of NanoVP in the blood and its subsequent anticancer performance.

The BBB limits the effective delivery of more than 98% of small‐molecule therapeutics to the brain.^[^ ^60^ ^]^ Many modalities have shown promise in permeabilizing the BBB, but are often challenged by edema and neurotoxicity.^[^ ^61^, ^62^, ^63^, ^64^, ^65^ ^]^ Several groups have shown the potential of light‐activated 5‐ALA (Gliolan)‐induced PpIX to open the BBB in rodents.^[^ ^66^, ^67^ ^]^ Hirschberg and colleagues showed larger doses of 5‐ALA‐PDT could open the BBB for up to 72 h,^[^ ^68^, ^69^ ^]^ but also found early signs of necrosis up to 5 mm away from the primary brain tumor. In addition, non‐tumor‐bearing animals that received 125 mg kg^−1^ 5‐ALA and 54 J experienced a 50% mortality rate within 5 days of treatment. We demonstrated that NanoVP‐PDT at subtherapeutic doses to achieve photodynamic priming^[^ ^36^, ^70^ ^]^ mediates BBB opening to increase model drug accumulation in the brain by 5.5‐fold compared to using 5‐ALA‐PDT. More importantly, we showed that low‐dose PDT using NanoVP did not result in healthy brain tissue damage, consistent with findings in low‐dose 5‐ALA‐PDT‐treated rats. At higher PDT doses, we found comparable neuropil damage in both NanoVP‐PDT and 5‐ALA‐PDT‐treated rats. Permeabilization may be mediated by changes in junctional protein phenotype as we observed in model systems,^[^ ^36^ ^]^ although further histological analysis is warranted to confirm mechanisms. Notably, it is difficult to compare PDT doses between two photosensitizers due to differing properties of the drug itself, differing temporal kinetics, and differing properties of the activating light. In this study, we compared 5‐ALA‐PDT at safe doses for BBB permeabilization as well as at clinically relevant doses for PDT. We found that NanoVP outperformed 5‐ALA under all conditions.

Intraoperative NanoVP‐PDT has a high translational potential. Surgery followed by PDT of GBM has been studied in the clinic (NCT03048240, NCT03897491, NCT00003788).^[^ ^71^, ^72^ ^]^ Despite promising results, the side effects of PDT remain a major concern.^[^ ^67^, ^69^, ^73^, ^74^, ^75^ ^]^ For example, we noted generation of heat in the laser fiber used for 635 nm activation of 5‐ALA. The photothermal effect may provide additional ablative power but may also present a serious complication for patients if neurological injury results. On the other hand, we observed no heat generation for 690 nm activation of NanoVP formulations, which is notable considering the advancement of 5‐ALA‐PDT in the clinic. The safest and most advantageous initial application of low‐dose NanoVP‐PDT may be intraoperative, where patients can receive PDT following tumor resection via fiber optic light conduits placed within the resection cavity.^[^ ^71^, ^72^ ^]^ We envision NanoVP‐PDT in this context, both sterilizing unresectable tumor cells during open surgery and permeabilizing the BBB for subsequent enhanced drug delivery. In rat brain tissues, we have shown that diffused light (690 nm) can reach up to 1.5‐2 centimeters deep to activate VP for effective PDT.^[^ ^9^ ^]^ This suggests that NanoVP‐PDT is sufficient to manage post‐surgical residual GBM cells that reside within 2 cm of the border of resection and that are responsible for ≈80% of recurrence. Moreover, the self‐limiting depth of effect avoids non‐specific priming of the underlying tissue. Beyond complementing surgical efforts, PDT has been successfully combined with chemotherapy, radiation, or immunotherapy due to differing mechanisms of action and their non‐overlapping side effects.^[^ ^76^ ^]^ Our next step is to investigate NanoVP‐PDT after primary tumor resection to open the BBB and enhance the subsequent delivery of chemotherapy or immunotherapy to invasive, microscopic cancer cells embedded within healthy brain tissues.

## 4 Experimental Section

### NanoVP Synthesis and Characterization

NanoVP was prepared using the solvent antisolvent precipitation method. Verteporfin (VP) powders (US Pharmacopeia) were dissolved in dimethyl sulfoxide (DMSO, solvent) to achieve different initial concentrations (1‐40 mM) and then injected dropwise into deionized water (antisolvent), under stirring (400 rpm, Thermo Scientific Cimarec Stirring Hot Plate), at room temperature. Different solvent‐antisolvent ratios (1:50‐1:4) were tested for formulating NanoVP. Samples were dialyzed (Spectrum Labs, MWCO 300 kDa) against phosphate‐buffered saline (PBS) at 4 °C for 24 h. The hydrodynamic diameter, polydispersity, and zeta potential were measured using a particle sizer and zeta potential analyzer (NanoBrook Omni, Brookhaven Instruments). VP concentration was determined based on UV‐Vis absorbance in DMSO (Synergy Neo2, BioTek Instruments) using the established molar extinction coefficient (VP: ε = 80 500 M^−1^ cm^−1^ at 435 nm, ε = 34 895 M^−1^ cm^−1^ at 687 nm). Entrapment efficiency is the percentage of VP successfully encapsulated into the NanoVP. Loading capacity is the amount of VP loaded per unit weight of the NanoVP. NanoSight tracking analysis (NanoSight LM10, Malvern Instruments) was used to determine the number of particles per milliliter. Liposomes are considered “gold standard” nanocarriers for VP delivery. Liposomes containing VP within phospholipid bilayers were synthesized via the freeze‐thaw extrusion technique (Supplementary Methods)^[^ ^76^ ^]^ and used as a control group. NanoVP synthesized using a 7 mM VP initial concentration and 1:50 DMSO:water ratio was used for the remaining experiments.

### Transmission Electron Microscopy (TEM)

The size, morphology, and microstructure of NanoVP were studied using TEM (JEOL JEM‐2100 LaB6, 200 kV). For conventional TEM, NanoVP (10 µL) was pipetted onto a Lacey carbon grid (Ted Pella) and air‐dried overnight before the examination. To exclude the possibility of artifacts due to sample dehydration, ionic liquid (3 µL, Hilem IL‐1000, Hitachi) was used as a pretreatment reagent for TEM examination of wet specimens. Images were taken at high magnifications (10 000–100 000X). Due to small particle sizes and to enhance diffraction intensity, selected area electron diffraction was used to collect electron diffraction patterns. For serum dissociation studies, NanoVP (final concentration 100 µM) was mixed with 1% or 10% v/v human serum (Fisher Scientific) for 2 h at 37 °C before drying on a Lacey carbon grid. Samples were imaged under the same conditions as pure NanoVP samples.

### NanoVP Stability, Photoactivity, and Photosensitizer Release Profile

The stability of NanoVP particles in PBS at 4 °C was determined by monitoring their hydrodynamic size and polydispersity index using a particle sizer analyzer for up to one year. NanoVP singlet oxygen (^1^O_2_) generation, self‐quenching, and drug release were studied in 96‐well plates as described previously.^[^ ^25^ ^]^ NanoVP and singlet oxygen sensor green (SOSG) at 5 µM were mixed and irradiated with 690 nm light (10 J cm^−2^, 10 mW cm^−2^; ML6600, Modulight). A multi‐mode microplate reader (Synergy Neo2, BioTek) acquired fluorescence signals of VP (Excitation/Emission: 435/650–750 nm) or SOSG (Excitation/Emission: 504/525 nm) before and after light irradiation. Self‐quenching (FL_DMSO_/F_PBS_) is defined as the fluorescence after disruption of the NanoVP using DMSO (FL_DMSO_) divided by the fluorescence of the NanoVP in PBS (FL_PBS_). Photosensitizer release from NanoVP (unquenching) was studied by monitoring the gain in VP fluorescence signal in PBS/DMSO buffer (0%–100% v/v) at room temperature or in Eagle's Minimum Essential Medium (EMEM, Cellgro) with 0%–10% v/v fetal bovine serum (FBS, Gibco) at 37 °C.

### Cell Cultures

The human glioblastoma U87 cell line and mouse 3T3 fibroblast cells were obtained from ATCC and cultured per the vendor's instructions. The human breast cancer MCF‐7 parental cell line, the P‐gp‐overexpressing MCF‐7 TX400 subline, and the ABCG2‐overexpressing MCF‐7 MX100 subline were cultured in EMEM supplemented with 10% FBS, 100 U mL^−1^ penicillin, 100 µg mL^−1^ streptomycin, and 0.01 mg ml^−1^ insulin (Sigma) as previously described.^[^ ^18^ ^]^ Cells were maintained in 5% CO_2_ at 37 °C and tested to be free of mycoplasma (MycoAlert, Lonza).

GBM39 cells expressing luciferase were serially passaged in the flank of female J:NU mice (4‐5 weeks old, #0 0 7850, Jackson Laboratory). Tumors were dissociated using a gentleMACS tumor dissociation kit (Miltenyi Biotec) before in vitro culturing. Cells were cultured as recommended by the Mayo Clinic Brain Tumor Patient‐Derived Xenograft National Resource using the FBS cell culture protocol. Briefly, cells were cultured in DMEM containing 2.5% FBS, 100 U mL^−1^ penicillin, and 100 µg mL^−1^ streptomycin for 48 h, then media was exchanged to DMEM containing 10% FBS and antibiotics for at least 24 h before experimentation. Cells were dissociated and used in experiments, but not passaged serially. Assays were performed within 14 days of tumor dissociation.

### Preparation of TBST VP

VP was dissolved in chloroform at a concentration of 250 µM and chloroform was evaporated under reduced pressure with a rotary evaporator. Tris‐buffered tween‐20 (TBST, 0.5%) was prepared by diluting a stock solution (ThermoScientific) with phosphate‐buffered saline (diluted to 1X from Calbiochem OmniPur 10X PBS (Millipore, 6507)). 1 mL of surfactant solution was added to a tube containing VP film and the samples were placed in a shaker at 37 °C at 100 rpm for 24 h. Samples were filtered using sterile syringe filters with 0.22 µm pore size (Millipore‐Sigma).

### Evaluation of Photosensitizer Uptake and PDT Responses In Vitro

Cells were cultured overnight in a 35‐mm Petri dish or 96‐well black wall plates (1–3.3 × 10^4^ cells cm^−2^) and then incubated with photosensitizers (i.e., NanoVP, free VP in DMSO, TBST VP, or liposomal VP at 0.25 µM; 5‐ALA at 5 mM) for 24 h for U87 cells or 1.5 h for GBM39 cells. Subsequently, cells were washed twice with PBS and incubated with a photosensitizer‐free complete medium. Photosensitizer uptake in cells was determined using extraction methods followed by VP fluorescence measurements (Excitation/Emission: 435/690 ± 20 nm, Synergy Neo2, BioTek) or visualized using fluorescence imaging (Lionheart, BioTek) as described previously.^[^ ^25^ ^]^ PDT was performed by exposing the cells to 690 nm light (0–10 J cm^−2^, 10–50 mW cm^−2^, bottom illumination; ML6600, Modulight). The generation of intracellular ROS was studied using 2′,7′‐dichlorofluorescin diacetate probe (DCFDA, Thermo Fisher) and the mitochondrial membrane potential was examined via TMRE assay (tetramethylrhodamine ethyl ester, Abcam). Expressions of total and cleaved caspase 3 were examined by immunoblotting (Supplementary Methods). At 24 h after PDT, cell viability was determined using MTT [3‐(4,5‐dimethylthiazol‐2‐yl)−2,5‐diphenyltetrazolium bromide] assay, neutral red assay (Abcam), or CellTiter Glo (Promega) following the vendor's protocol. For dark toxicity evaluation, cells were incubated with media containing photosensitizers (0–40 µM) for 72 h, followed by the MTT assay. The photosensitizer efflux by ATP‐binding cassette (ABC) transporters was studied in MCF‐7 and its multi‐drug resistant sublines (MCF‐7 TX400 and MCF‐7 MX100) by adapting our protocol (Supporting Methods).

### In Vivo Photodynamic Therapy and Photosensitizer Biodistribution

Animal protocols (R‐MAR‐22‐16, R‐MAY‐20‐19, R‐MAY‐23‐27) were approved by the University of Maryland, College Park Institutional Animal Care and Use Committee, which has been fully accredited by the Association for Assessment and Accreditation of Laboratory Animal Care International (AAALAC) since 2011 with PHS Assurance Number D16‐00172 and USDA Certificate Number 51‐R‐0095. Xenograft mouse models of glioblastoma were established by subcutaneously injecting U87 cells (1 × 10^6^ cells in PBS/Matrigel) into the flank of a J:NU mouse (4‐5 weeks old, Jackson Laboratory). Tumor volumes were longitudinally monitored using calipers and calculated using the standard estimation formula, V = ½ × length × width^2^, where length equals the maximum tumor diameter in millimeters and width equals the diameter that is perpendicular to the length. Treatments were initiated 2 weeks post‐implantation when tumors reached ≈100 mm^3^. At 2 h post‐IV injection of photosensitizers (i.e., NanoVP or liposomal VP; 0.5 mg kg^−1^) or PBS, a vertical 690 nm laser beam (100 J cm^−2^, 100 mW cm^−2^; ML6600, Modulight) was focused on the tumor to activate PDT. A cloth was used to protect animal skin from light exposure. Change in tumor volume was monitored for up to 2 months. The specific growth rate (SGR) of tumors was estimated using the equation (1/V)(dV/dt), where *V* is tumor volume and *t* is time. To examine photosensitizer biodistribution, tumor and normal tissues were collected at 2‐ and 24 h post‐injection of photosensitizers.

GBM39 cells were passaged and dissociated as described above. 5 × 10^5^ GBM39 cells in 5 µL of PBS were stereotactically injected using a Hamilton syringe positioned at 2 mm right lateral to bregma and 3 mm deep in female J:Nu mice (4‐5 weeks old, #0 0 7850, Jackson Laboratory). Fourteen days post‐implantation, PDT was performed. Mice were randomly divided into NanoVP‐PDT, liposomal VP‐PDT, TBST VP‐PDT, 5‐ALA‐PDT and no treatment groups. Mice received 0.5 mg kg^−1^ tail vein injections of NanoVP, TBST VP, or liposomal VP and, after 2 h, received 12 J cm^−1^ of 690 nm light (40 mW cm^−1^) interstitially through the burr hole used to implant tumor cells (RD10 laser fiber, ML6600 laser, Modulight). Mice treated with 5‐ALA PDT received 20 mg kg^−1^ 5‐ALA IV 4 h before laser irradiation with 635 nm light (100 mW cm^−1^, 60 J cm^−1^). Power level was measured using the Modulight MLACAL external calibration unit. All but 5 mm from the tip of the RD10 fiber, matching the depth that the fiber penetrated the mouse brain, was covered with black masking tape (Thor Labs) before measuring power according to manufacturer instructions.

### Photodynamic Opening of the Blood‐Brain Barrier in Rodents

Animal protocols were approved by the University of Maryland, School of Medicine Institutional Animal Care and Use Committee. Sprague‐Dawley rats (4‐5 weeks old, Envigo) received an IV injection of NanoVP (0.25 or 0.5 mg kg^−1^) or 5‐aminolevulinic acid (5‐ALA, 20 or 125 mg kg^−1^) at 30 min or 4 h before PDT. PDT parameters, including photosensitizer concentration, drug‐light interval, irradiance, and radiant exposure, were selected based on our experience or others’ clinical work. At 30 min post‐photosensitizer injection, rats were anesthetized with isoflurane, secured within a stereotaxic frame, and a craniotomy was performed to expose the right cerebral hemisphere (Supporting Methods). PDT was performed by light activation of the exposed brain (NanoVP: 690 nm, 80 or 100 J cm^−2^; 5‐ALA: 635 nm, 60 or 80 J cm^−2^; 40 or 85 mW cm^−2^; ML6600, Modulight). At 90 minutes post‐PDT, rats received an IV injection of Evans blue dye (2%, 4 mL kg^−1^) to determine the blood‐brain barrier integrity using imaging and extraction methods (Supporting Methods). In a small group of PDT animals that did not receive Evans blue injection, brain tissues were collected, sectioned, and processed for i) histological (hematoxylin and eosin stain, H&E) analysis and ii) Luxol Fast Blue staining of myelin/myelinated axons and Nissl bodies. H&E and immunohistochemistry slides were imaged using the whole slide Aperio AT2 scanner system (Leica Biosystems) (Supplementary Methods). All image analysis was accomplished using Aperio and Halo imaging analysis software (v3.3.2541.300; Indica Labs), and image annotations were performed by a pathologist (B.K). Fields were excluded if they contained large areas of artifact such as folds or tears.

### Statistical Analyses

Results are presented in mean ± standard error of the mean (SEM). Statistical tests were carried out using GraphPad Prism (GraphPad Software). Specific tests and the number of repeats are indicated in the figure captions. Reported P values are two‐tailed. One‐way or two‐way ANOVA statistical tests with Tukey post‐hoc tests were performed.

### IVIS Imaging

IVIS bioluminescent in vivo imaging (PerkinElmer) and LivingImage software were used to measure tumor burden over time in the orthotopic GBM model. Mice received 150 mg kg^−1^ intraperitoneal injection of luciferin (Promega VivoGlo) ten minutes before imaging, then were anesthetized and imaged. Regions of interest of equal size were used to quantify total flux (photons/second).

### Pharmacokinetics and Quantitative Biodistribution

For pharmacokinetic analysis, a mixed cohort of male and female J:Nu mice bearing flank GBM39 tumors (1 × 10^6^ cells in PBS/Matrigel) measured to 50 mm^3^, as described above, received 0.5 mg kg^−1^ tail vein injections of NanoVP. After euthanasia at 0.05, 0.25, 1, 3, 6, 24, or 72 h post‐injection, blood was collected in lithium heparin tubes (Sarstedt, Inc.) and centrifuged at 2000xg. Serum was mixed 1:10 with HPLC‐grade acetonitrile, stored at −80 °C for 1 h, then centrifuged again at 10 000 rpm for 20 min at 4 °C. Supernatants were collected for HPLC/MS‐MS analysis. Tissues were stored at −80 °C until processed. Tissues were homogenized with a BeadBug 6 (Benchmark Scientific) and 2.0 mL tubes prefilled with 3.0 mm zirconium homogenizer beads (Stellar Scientific). Tissues were mixed 1:3 w/w with HPLC‐grade water (Sigma–Aldrich) for homogenization, then mixed 1:8:1 sample:acetonitrile:methanol v/v. Samples were stored at −80 °C for at least 30 min before centrifugation at 21.1xg for 20 min. Supernatants were transferred to centrifugal polytetrafluoroethylene filters (MilliposeSigma), centrifuged according to manufacturer protocol, and then transferred to HPLC tubes (Waters). Waters H‐Class UPLC/Xevo TQD mass spectrometer was used for quantification of VP in both serum and tissues. Samples and standards were analyzed using an ACQUITY UPLC BEH reversed phase C18 column (130 Å, 1.7 µm, 2.1 mm ×  50 mm, Waters). At a flow rate of 0.4 mL min^−1^, liquid chromatography was performed where A was water with 0.1% formic acid and B was acetonitrile with 0.1% formic acid: 20% A from 0–1 min; 20% A to 0% A from 1–3.2 min; 0% A to 20% A from 3.2–3.3 min; 20% A from 3.3 to 3.5 min. VP eluted in two peaks at ≈3.02 and ≈3.11 min. Daughter ions detected were 645.45, 513.37, and 499.93 m z^−1^; the 513.37 ion was used for quantitation.

## Conflict of Interest

The authors declare no conflict of interest.

## Author Contributions

J.A.Q. and C.T.I. contributed equally to this work. C.I., and H.C.H. performed conceptualization; C.I., J.A.Q., W.A.C., and B.K. performed methodology; C.I., J.A.Q., P.S., I.R., J.S., C.A., K.B., A.G., B.G., W.A.C., B.K., N.C., and H.C.H. performed investigation; C.I., J.A.Q., and H.C.H. performed visualization; M.M.G., and H.C.H. managed funding acquisition; H.C.H. managed project administration; M.M.G., and H.C.H. performed supervision; C.I., H.C.H. wrote the original draft; J.A.Q., C.I., P.S., I.R., J.S., C.A., K.B., A.G., B.G., P.S., W.A.C., B.K., N.C., R.R., G.F.W., M.M.G., and H.C.H. wrote, reviewed and edited the manuscript.

## Supporting information

Supporting Information

## Data Availability

The data that support the findings of this study are available in the supplementary material of this article.
