# Supplementary material for: Carrier‐Free, Amorphous Verteporfin Nanodrug for Enhanced Photodynamic Cancer Therapy and Brain Drug Delivery
Source: Adv Sci (Weinh). 2024 Mar 6;11(17):2302872. doi: 10.1002/advs.202302872 (PMC11077681; doi:10.1002/advs.202302872)
Supplement: Supplementary file 1 — Supporting Information [file ADVS-11-2302872-s001.pdf]

ADVANCED SCIENCE
Open Access

Supporting Information

for *Adv. Sci.*, DOI 10.1002/advs.202302872

Carrier-Free, Amorphous Verteporfin Nanodrug for Enhanced Photodynamic Cancer Therapy and Brain Drug Delivery

*John A. Quinlan, Collin T. Inglut, Payal Srivastava, Idrisa Rahman, Jillian Stabile, Brandon Gaitan, Carla Arnau Del Valle, Kaylin Baumiller, Anandita Gaur, Wen-An Chiou, Baktiar Karim, Nina Connolly, Robert W. Robey, Graeme F. Woodworth, Michael M. Gottesman and Huang-Chiao Huang**

List of Supplementary Materials

Supplementary Materials and Methods

1. *Liposomal VP synthesis and characterization*
2. *NanoVP electrostatic stabilization*
3. *Intracellular ROS production using DCFDA*
4. *Mitochondrial membrane potential using TMRE*
5. *Caspase-3 western blots*
6. *Drug efflux assay*
7. *Photosensitizer biodistribution analysis*
8. *Rat craniotomy*
9. *Evans Blue quantification*
10. *Brain histology*

Supplementary Figure S1. Size quantification of NanoVP TEM micrograph images.

Supplementary Figure S2. Representative ionic liquid TEM micrograph.

Supplementary Figure S3. Alternative NanoVP synthesis methods.

Supplementary Figure S4. NanoVP diameter.

Supplementary Figure S5. NanoVP shelf life.

Supplementary Figure S6. NanoVP shelf life under differing storage conditions.

Supplementary Figure S7. Probing NanoVP electrostatic stabilization.

Supplementary Figure S8. High power light activation of VP results in limited photothermal effects.

Supplementary Figure S9. PDT efficacy within 3T3 fibroblast cells.

Supplementary Figure S10. VP-mediated PDT does not induce lysosomal damage.

Supplementary Figure S11. NanoVP is a substrate for ABC transporters ABCG2 and P-gp.

Supplementary Figure S12. VP biodistribution in mice organs and U87 tumors.

Supplementary Figure S13. Mouse weight post-PDT.

Supplementary Figure S14. Formulation of molecularly dispersed VP control.

Supplementary Figure S15. Mouse weight with intracranial tumor injection and interstitial PDT.

Supplementary Figure S16. PDT with Liposomal VP, TBST VP, or NanoVP does not generate heat *in vivo*, but the fiber used for 5-ALA PDT does generate heat.

Supplementary Figure S17. Traditional high-dose PDT damages healthy brain tissue.

Supplementary Table S1. Biodistribution of NanoVP.

Supplementary Table S2. Parameters in clinical applications of 5-ALA PDT.

Supplementary Table S3. Summary of studies establishing blood-brain barrier permeabilization parameters.

Supplementary Materials and Methods

1. Liposomal VP synthesis and characterization

Liposomes containing verteporfin (VP) within phospholipid bilayers were synthesized via the freeze-thaw extrusion technique and characterized following our protocol (*1*). Briefly, dipalmitoylphosphatidylcholine (DPPC), cholesterol, distearoylphosphatidylethanolamine-methoxy polyethylene glycol (DSPE-mPEG2000), and dioleoyltrimethylammoniumpropane (DOTAP; Avanti Polar Lipids) were mixed in chloroform at 6:3:0.3:0.7 molar ratio and co-dissolved with 50-200 nanomoles of VP (US Pharmacopeia). Chloroform was removed by a rotary evaporator to create a thin lipid film, which was rehydrated with 1 mL of ultrapure deionized water (Invitrogen). The phospholipid VP suspension was subjected to freeze-thaw cycles (4-45°C). The dispersions were extruded through polycarbonate membranes (0.1 µm pore size; Avanti Polar Lipids) at 45°C to form unilamellar vesicles. Liposomal VP samples were dialyzed against 1X phosphate-buffered saline (PBS) at 4°C and stored at 4°C until use. The concentrations and loading capacity of liposomal VP were determined by UV-Vis spectroscopy. Hydrodynamic diameter, polydispersity, and zeta potential of liposomal VP were measured using a particle sizer and zeta potential analyzer (NanoBrook Omni, Brookhaven Instruments).

2. NanoVP electrostatic stabilization

The electrostatic stability of NanoVP was probed using the neutral polymer, poloxamer 407 (Pluronic ® F-127; Sigma), and the cationic polymer, polyethylenimine (PEI). Polymers were dissolved in water according to the manufacturer's instructions and were added to the antisolvent (ultra-pure deionized water) prior to drop-wise synthesis or after NanoVP particles were formed, at a 1:5 polymer: VP ratio (w:w). In both instances, the addition of positively charged PEI resulted in uncontrollable agglomeration (**Supplementary Fig. 6**), while NanoVP particles coated with poloxamer 407 appeared to remain stable. Dynamic light scattering (DLS) and laser Doppler electrophoresis (LDE) measurements showed that NanoVP coated with poloxamer 407 has a hydrodynamic diameter of 112.7±5.9 nm (PdI: 0.10±0.01) and zeta potential of -32.6±1.8 mV. DLS and LDE measurements cannot be accurately performed on large agglomerations.

3. Intracellular ROS production using DCFDA

Intracellular reactive oxygen species (ROS) production was examined via 2',7'–dichlorofluorescin diacetate (DCFDA; Thermo Fisher) assay. U87 cells were plated in 96-well black wall plates (Krystal) at a density of 2.2×10^4 cells/cm^2. On the next day, cells were incubated with complete culture medium containing 0.25 µM of NanoVP, free-form VP, or Liposomal VP for 24 hours. On day 3, prior to photodynamic therapy (PDT), cells were washed twice with PBS and incubated with 10 µM DCFDA for 30 minutes. Cells were irradiated with 690-nm red light (0-10 J/cm^2, 50 mW/cm^2; ML6600, Modulight). For the positive control, cells were incubated with 0.1 mL of 100 µM H_2O_2 for 15 minutes. The fluorescence signal of the cleaved DCF probe (Excitation/Emission: 485/535±15 nm) was measured using a microplate reader (Synergy Neo2, BioTek).

4. Mitochondrial membrane potential using TMRE

Mitochondrial membrane potential (ΔΨm) was examined via tetramethylrhodamine ethyl ester (TMRE; Abcam) assay. U87 cells were plated in 96-well black wall plates at a density of 2.2×10^4 cells/cm^2. After a 24-hour incubation with 0.25 µM of NanoVP, free-form VP, or Liposomal VP, cells were washed twice with PBS and exposed to 690-nm light (0-10 J/cm^2, 50 mW/cm^2). After PDT, cells were incubated with 250 nM TMRE for 25 minutes and washed twice with 0.2% bovine serum albumin (BSA) within PBS. Samples were subjected to fluorescence readings (Excitation/Emission: 549/575±10 nm) using a microplate reader or fluorescence and phase contrast imaging (Lionheart, BioTek). Controls include no light irradiation and incubation with 100 µM of carbonilcyanide p-triflouromethoxyphenylhydrazone (FCCP), a mitochondrial uncoupler, for 25 minutes. PDT and FCCP-treated samples were normalized to a no-treatment control.

5. Caspase-3 western blots

Expression of total and cleaved caspase-3 were examined using immunoblotting techniques. U87 cells were plated in 35-mm Petri dish (Falcon) at a density of 2.2×10^4 cells/cm^2. Cells were incubated with 0.25 µM of NanoVP, free-form VP, or liposomal VP for 24 hours. Cells were washed twice with PBS and irradiated with 690-nm light (10 J/cm^2, 50 mW/cm^2) in fresh culture medium. At 1 hour after PDT, cell lysates were collected in radioimmunoprecipitation assay (RIPA) buffer supplemented with 1% protease inhibitor. Protein lysates (22 µg) were separated on a 4-12% precast Bis-Tris gel (NuPAGE) and transferred onto a nitrocellulose membrane. After blocking with Odyssey blocking buffer (Li-COR) for 1 hour, blots were incubated with primary antibody against beta-actin (#3700, cell signaling), caspase 3 (#9662, Cell Signaling,), or cleaved caspase 3 (#9661, Cell Signaling) overnight at 4 °C. Blots were washed with 1X Tris-Buffered Saline, 0.1% Tween® 20 Detergent (TBST) buffer followed by incubation with IRDye 680RD goat anti-mouse secondary antibody (926-68070, Li-COR) or IRDye 800CW goat anti-rabbit secondary antibody (926-32211, Li-COR) for 1 hour at room temperature based on the supplier's advice. Visualization of protein bands was developed using the LiCor ODYSSEY CLx (Li-COR). β-actin serviced as a loading control. Band intensity was determined using LiCOR ODYSSEY CLx software. PDT treated samples were normalized to a no treatment control.

6. Drug efflux assay

To study ATP-binding cassette (ABC) transporter-mediated photosensitizer efflux, MCF-7, MCF-7 TX400, or MCF-7 MX100 were plated in 35 mm Petri dishes (Falcon) at a density of 3.3 x10^4 cells/cm^2. The next day, cells were incubated with 1 µM of NanoVP, free-form VP, or liposomal VP for 4 hours at 37 ºC with or without ABC drug transporter inhibitors (ABCG2 inhibitor: 10 mM fumetrimorgin C; P-gp inhibitor: 2.5 mM tariquidar). Subsequently, cells were washed twice with PBS, trypsinized, and lysed in RIPA buffer at 4 ºC. VP fluorescence (Excitation/Emission: 435/690 nm) was measured using a multi-mode microplate reader, and intracellular VP concentration was determined with appropriate VP standard curves. Intracellular VP was normalized to total cell protein. The total protein amount was determined using the Pierce BCA protein assay (Thermo Fisher).

7. Rat craniotomy

Rats were anesthetized with isoflurane and secured within a stereotaxic frame. During the 30-minute drug-light interval, a craniotomy was performed. The top of the rat head was shaved, and the skin was removed. The periosteum was retracted to the edges of the skull. Using a micro drill (OmniDrill35, WPI), a circle about 4 mm in diameter was sketched into the right parietal bone. Drilling was stopped periodically, and PBS was applied to prevent heating. The drilled bone was removed, and any bleeding that occurred from surgery was stopped prior to light irradiation. After a 30-minute circulation period with Evans blue, rats were sacrificed via isoflurane overdose and decapitation, and brains were harvested. Digital images were immediately captured, and the brains were frozen at -80°C.

8. Evans Blue quantification

Evans blue was quantified as previously described (*2*). Briefly, the brain hemispheres were thawed on ice, minced, and incubated with formamide for 30 minutes at 55°C followed by 4 days at room temperature. Samples were centrifuged to pellet the tissue, and the optical density of Evans blue within the supernatant at 610 nm was measured on a multi-mode microplate reader. Standard curves were used to determine the amount of Evans Blue in each brain hemisphere.

9. Brain histology

The effects of PDT on healthy brain tissues were examined 2 hours after light irradiation. Brains were harvested and frozen within optimum cutting temperature (OCT) compound (Tissue-Tek) at -80°C. Cryocut sections (5 µm) from frozen rat brains were collected at the level of rostral and caudal to the treatment area and fixed in a mixture of glutaraldehyde and formaldehyde. Hematoxylin and eosin (H&E) staining was performed using the Sakura® Tissue-Tek® Prisma™ automated stainer. The slides were hydrated and stained with commercial hematoxylin, clarifier, bluing reagent and eosin-Y. A regressive staining method was used. This method intentionally overstains tissues and then uses a differentiation step (clarifier/bluing reagents) to remove excess stain. The slides were cover slipped using the Sakura® Tissue-Tek™Glass® automatic cover slipper and dried prior to review. For Luxol Fast Blue staining, sections were hydrated and stained overnight at room temperature. Sections were immersed in 95% alcohol to remove excess stain. Slides were then washed in distilled water and quickly immersed in 0.05% lithium carbonate. Tissue sections were then placed in 70% ethanol until a sharp contrast between the corpus callosum and cortex developed. Sections were washed in distilled water, stain with cresyl violet acetate, differentiate in several changes of 95% alcohol, dehydrated with ethanol, cleared in xylene, and mounted. Slides were scanned at 20X using an Aperio AT2 scanner (Leica Biosystems) into whole slide digital images.

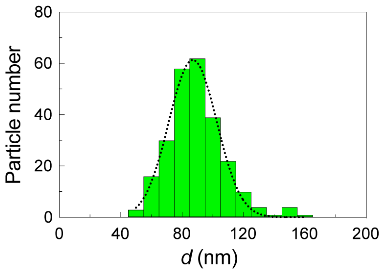

Supplementary Figure S1. Size quantification of NanoVP TEM micrograph images. Black line shows the gaussian distribution curve of the data set. Bin size = 10 nm. N = 250 particles.

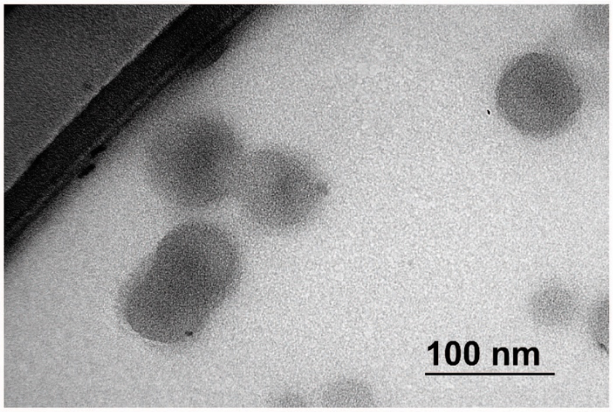

Supplementary Figure S2. Representative ionic liquid TEM micrograph. A second electron imaging technique was used to verify the ‘drying effect’ did not impact NanoVP structure or morphology. Scale bar = 100 nm.

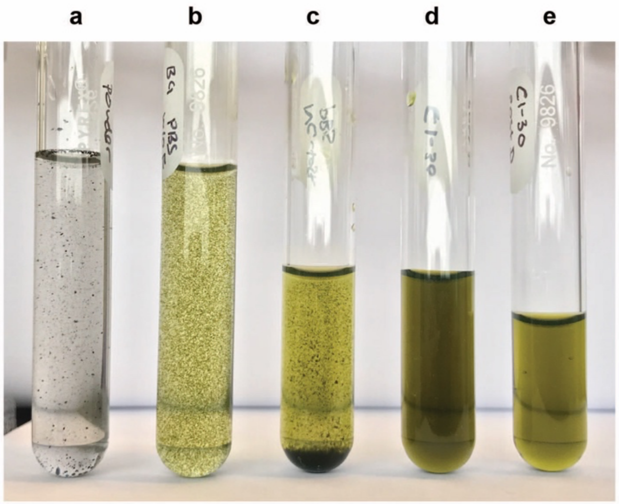

Supplementary **Figure S3. Alternative NanoVP synthesis methods.** Representative digital images of (**a**) VP lyophilized powder was added to PBS and did not dissolve. Fully dissolved VP in DMSO was added into PBS (**b**) dropwise and (**c**) non-dropwise. Adding VP solution directly into PBS results in uncontrolled VP aggregation. (**d**) NanoVP synthesis following established preparticipation method in water. (**e**) NanoVP post dialysis in PBS.

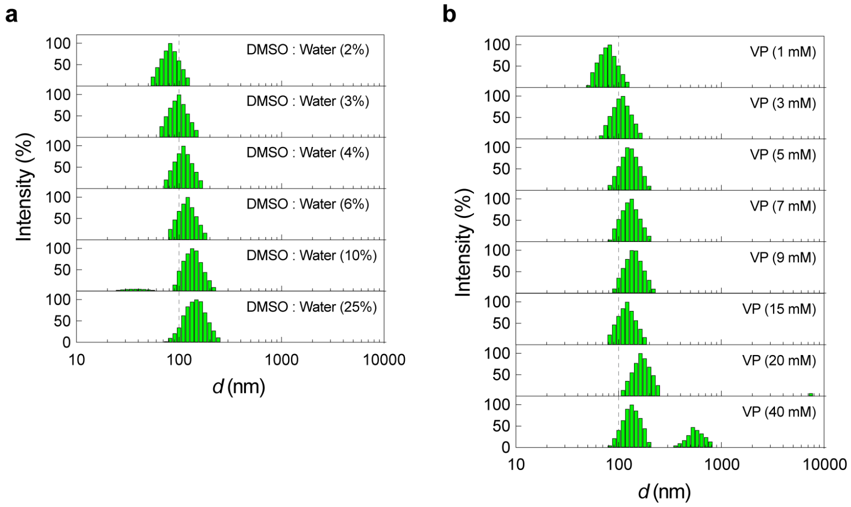

Supplementary Figure S4. NanoVP diameter. Representative intensity plots of NanoVP as a function of (**a**) DMSO: Water ratio and (**b**) initial VP concentration.

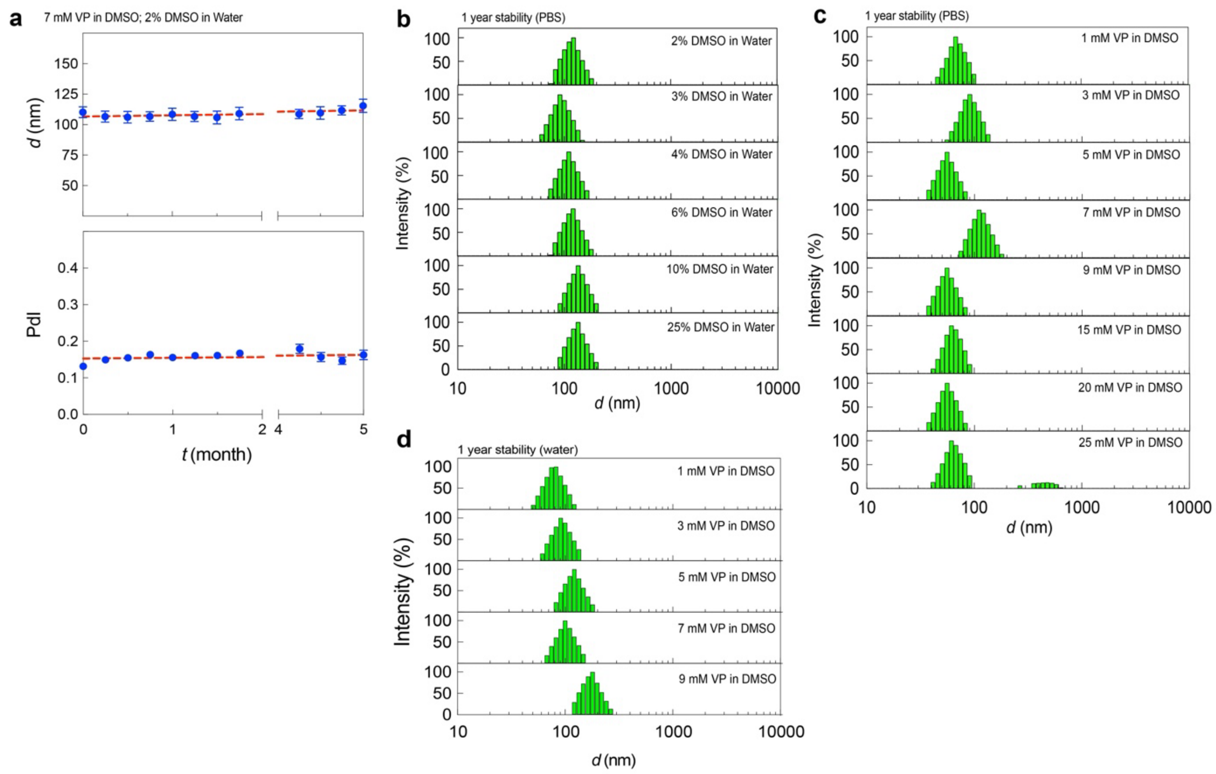

Supplementary Figure S5. NanoVP shelf life. (**a**) Standard (7 mM VP in DMSO, 2% DMSO:water ratio) NanoVP diameter and PdI dialyzed into PBS and representative intensity plots of (**b**) varying DMSO:water ratios dialyzed into PBS, **(c)** varying VP in DMSO concentrations dialyzed into PBS, and (**d**) varying VP in DMSO concentrations in water.

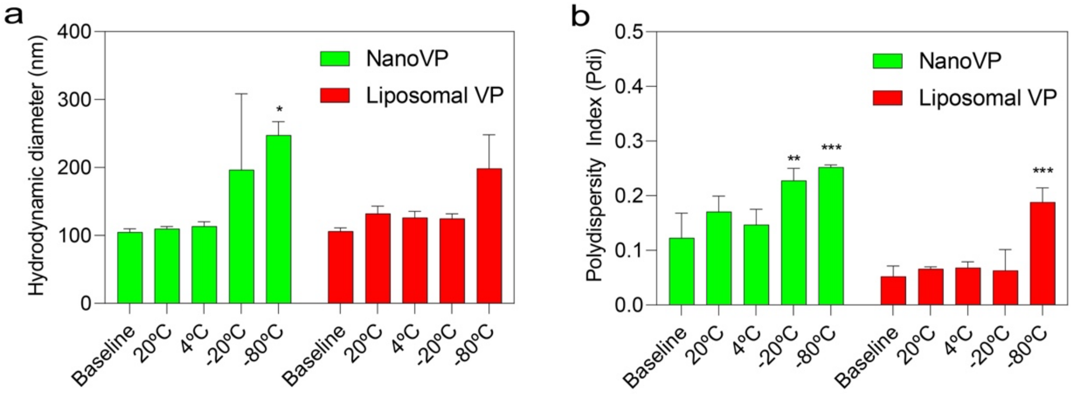

Supplementary Figure S6. NanoVP shelf life under differing storage conditions. NanoVP made under standard conditions (7 mM VP in DMSO, 2% DMSO:water ratio) were stored for 90 days at room temperature, 4°C, -20°C, and -80°C. (**a**) Hydrodynamic diameter and (**b**) polydispersity index at baseline (immediately after formulation) were compared to values after storage. Two-way ANOVA with multiple comparison test was used to calculate significant differences, where * $P < 0.05$, ** $P < 0.01$, *** $P < 0.001$. Error bars show standard errors of the mean.

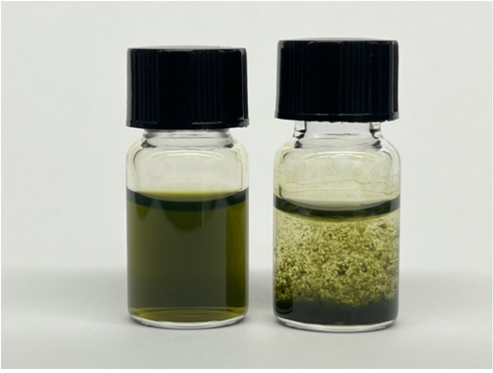

Supplementary Figure S7. Probing NanoVP electrostatic stabilization. Representative digitals images of NanoVP coated polymers. NanoVP was synthesized with (**left**) neutral polymer poloxamer 407 or (**right**) cationic polymer polyethylenimine. Poloxamer 407 had no impact on NanoVP stability, size, nor PdI. Polyethylenimine caused NanoVP to form large aggregates.

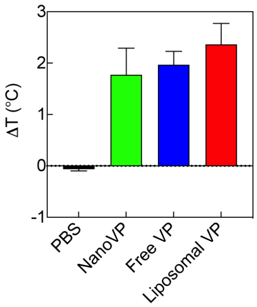

Supplementary Figure S8. High-power light activation of VP results in limited photothermal effects. NanoVP, free-form VP, or Liposomal VP (40 μM) in PBS were light-activated (50 J/cm^2, 0.5 W/cm^2) and solution temperature was measured. Error bar shows the standard error of the mean.

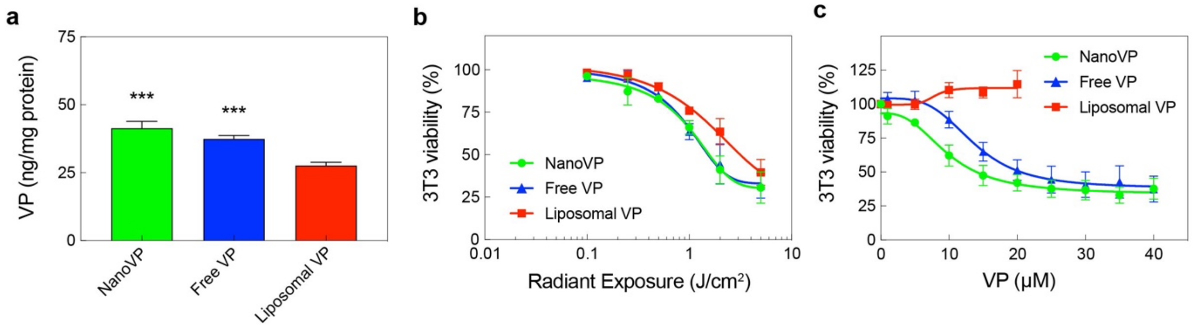

Supplementary Figure S9. PDT efficacy within 3T3 fibroblast cells. (**a**) The intracellular VP concentration was determined via extraction methods at 24 hours post incubation with 0.25 μM of NanoVP, free-form VP, or Liposomal VP. (**b**) Cell viability measured via MTT assay 24 hours after NanoVP, free-form VP, or Liposomal VP-mediated PDT (690-nm, 0-5 J/cm^2, 10 mW/cm^2). (**c)** Cell viability was measured via MTT assay 72 hours after incubation without light activated of NanoVP, free-form VP, or Liposomal VP. One-way ANOVA with multiple comparison test was used to calculate significant differences, where *** $P < 0.001$. Error bars show standard errors of the mean.

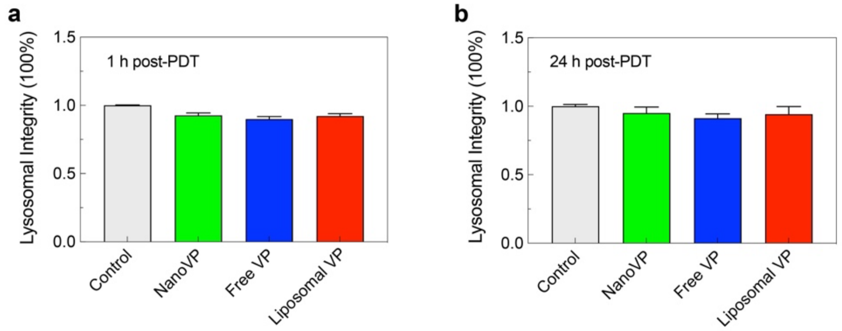

Supplementary Figure S10. VP-mediated PDT does not induce lysosomal damage. Lysosomal damage was assessed via NRU assay (**a**) 1-hour and (**b**) 24-hours post-PDT (1 J/cm^2, 50 mW/cm^2) of 0.25 μM NanoVP, free-form VP, or Liposomal VP. One-way ANOVA with multiple comparison test was used to calculate significant differences, where * $P < 0.05$, N ≥ 3. Error bars show standard errors of the mean.

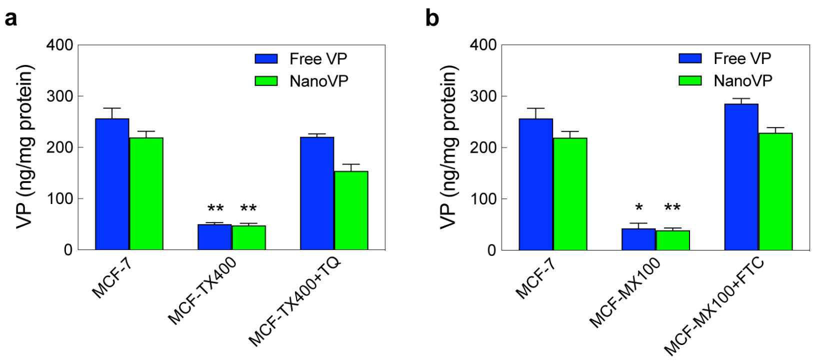

Figure S11. NanoVP is a substrate for ABC transporters ABCG2 and P-gp. Intracellular VP concentration was determined via extraction methods at 4-hours post-incubation with 1 µM NanoVP or free VP. Breast cancer cells overexpressing (**a**) P-gp (MCF-7 TX400) or (**b**) ABCG2 (MCF-7 MX100), and parental MCF-7 cells were incubated with and without inhibitors (10 µM FTC for ABCG2 or 2.5 mM tariquidar for P-gp). Two-way ANOVA with multiple comparison test was used to calculate significant differences, where * $P < 0.05$, ** $P < 0.01$, $N \geq 3$. Error bar shows the standard error of the mean.

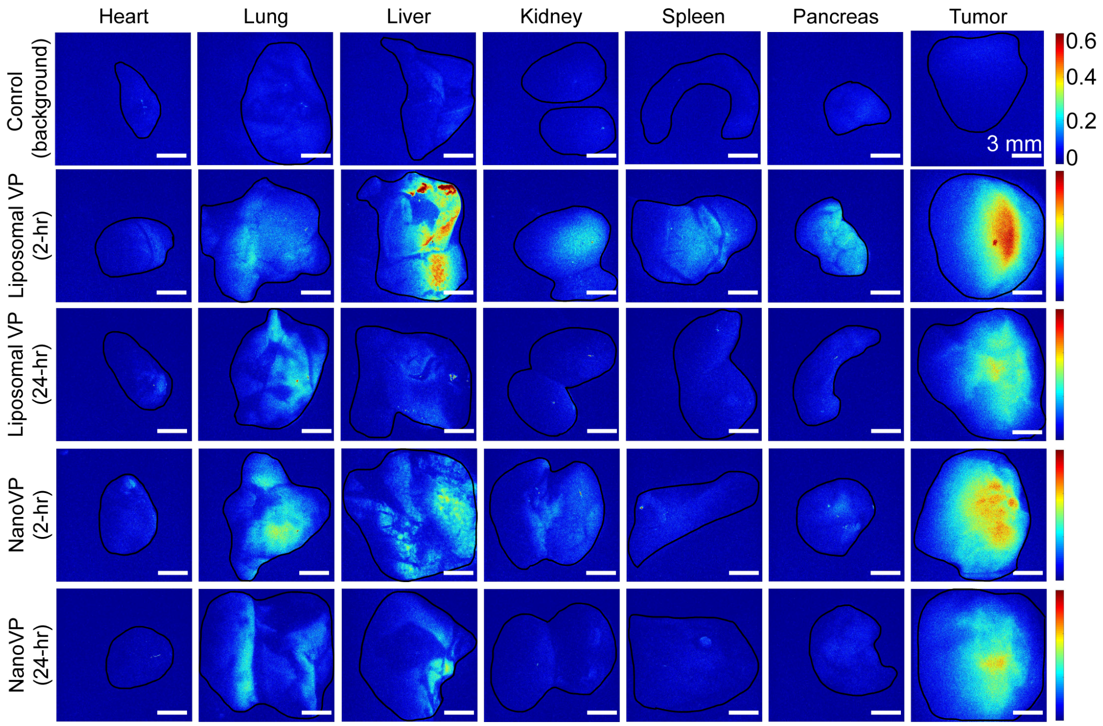

Figure S12. VP biodistribution in mice organs and U87 tumors. Representative fluorescence images of NanoVP and Liposomal VP in mice organs and U87 tumors at both 2 hours and 24 hours post IV injection. Scale bar = 3 mm.

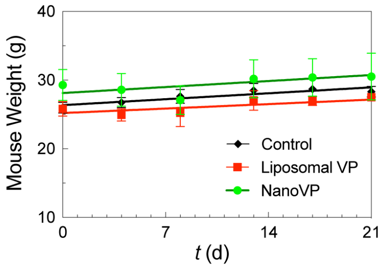

Figure S13. Mouse weight post-PDT. PDT had no impact on weight.

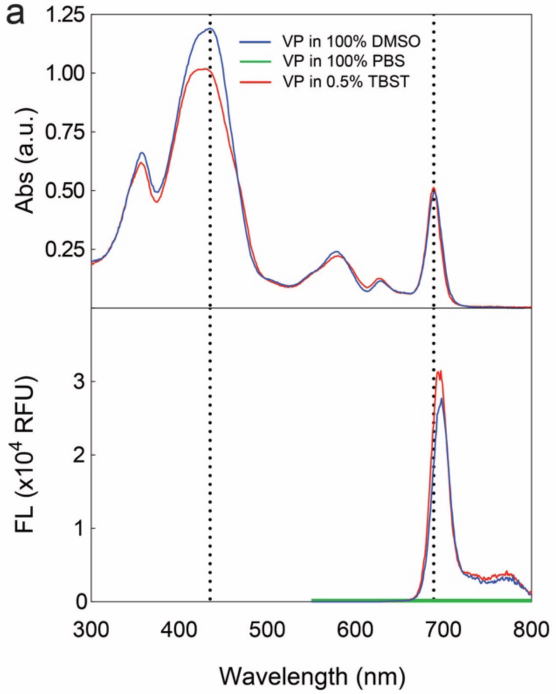

b

Surfactant (%)	Abs at 690 nm (%)	FL at 690 nm (%)
DMSO (100)	100.0	100.0
PBS (100)	1.4	0.0
Tris-buffered Tween-20 (0.5)	102.4	133.0

Supplementary Figure S14. **Formulation of molecularly dispersed VP control**. VP is fully soluble in DMSO but not soluble in PBS; as such, absorbance and fluorescence are at a maximum in DMSO and minimum in PBS. (**a**) Absorption and fluorescence spectra (excitation: 435 nm) reveal that VP in 0.5% tris-buffered tween-20 (TBST) is fully unquenched and molecularly dispersed, as quantified in (**b**). No particles were observed on DLS and the resulting mixture had VP dispersed throughout.

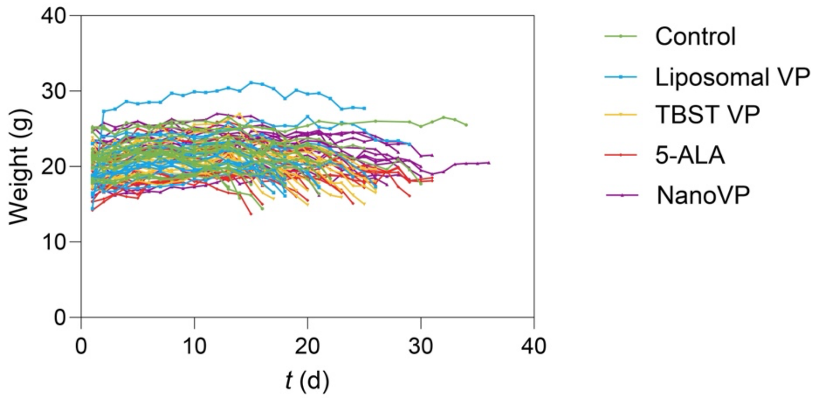

Supplementary Figure S15. Mouse weight with intracranial tumor injection and interstitial PDT. Mouse weight was minimally impacted by either procedure, although weight did decline as tumor burden increased.

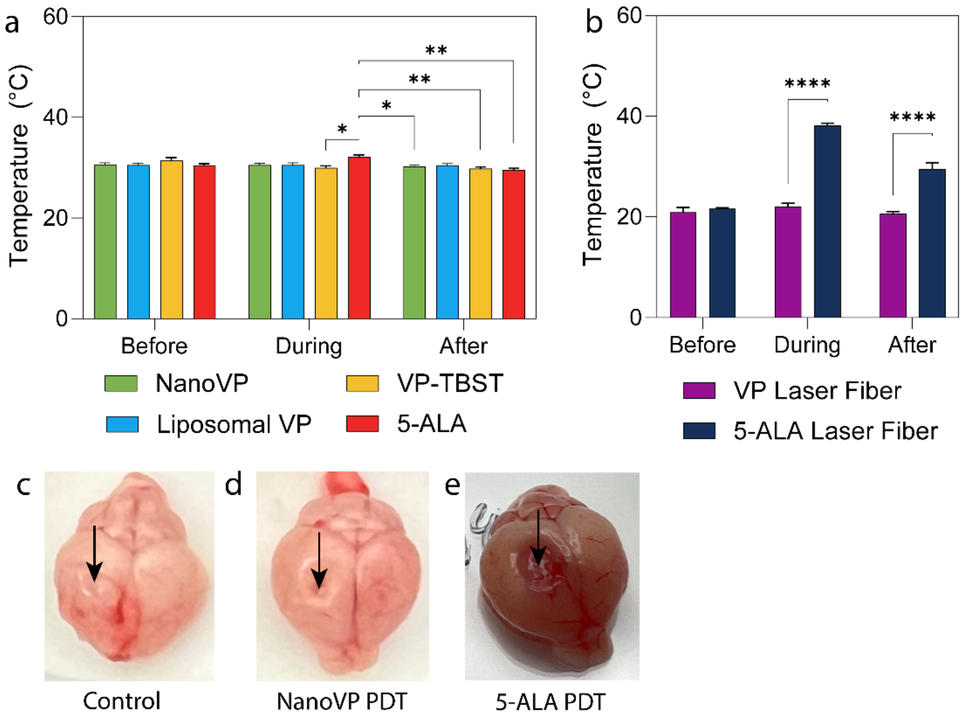

Supplementary Figure S16. PDT with Liposomal VP, TBST VP, or NanoVP does not generate heat *in vivo*, but the fiber used for 5-ALA PDT does generate heat. (**a**) A FLIR infrared camera was used to record temperature at the site of PDT as well as (**b**) the laser fiber before, halfway through, and immediately after PDT. A black arrow indicates the site of tumor cell injection and laser fiber insertion in (**c**) Control brains, (**d**) NanoVP-PDT treated brains, and (**e**) 5-ALA PDT treated brains, with an apparent ablation zone around fiber insertion site only in 5-ALA PDT treated brains at endpoint (approximately two weeks post-PDT). N ≥ 3 for all groups. By two-way ANOVA with Tukey's multiple comparison test, * $P < 0.05$, ** $P < 0.01$, **** $P < 0.001$. Data presented as mean ± SEM.

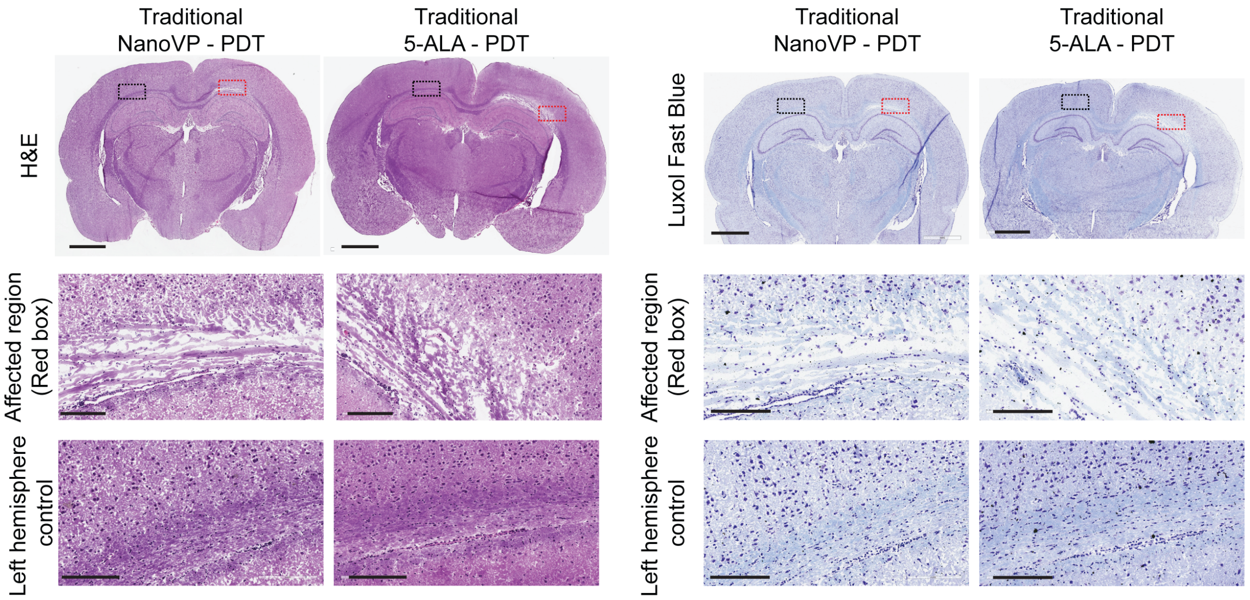

Figure S17. Traditional high-dose PDT damages healthy brain tissue. Representative photomicrographs of brain sections stained with H&E and Luxol fast blue. Photosensitizers (0.5 mg/kg NanoVP or 125 mg/kg 5-ALA) were photoactivated (NanoVP: 690 nm, 100 J/cm^2, 40 mW/cm^2; 5-ALA: 635 nm, 60 J/cm^2, 40 mW/cm^2) 30-minutes (for NanoVP) or 4-hours (for 5-ALA) after IV injection. Brains were harvested 90 minutes post-PDT.

Supplementary Table S1. Biodistribution of NanoVP. Mice received IV injection of NanoVP (0.5 mg/kg) and tissues were homogenized before quantification of VP in each tissue by HPLC-MS/MS. Values shown are percent injected dose per organ (%ID/organ) ± standard error of the mean.

	**0.25 h**	**1 h**	**3 h**	**6 h**	**24 h**	**72 h**
**Heart**	0.01 ± 0.01	0.47 ± 0.33	3.65 ± 2.10	16.9 ± 7.43	2.07 ± 0.83	0.14 ± 0.08
**Eyes**	0.00 ± 0.00	0.03 ± 0.02	0.06 ± 0.02	0.31 ± 0.11	0.84 ± 0.34	0.12 ± 0.06
**Lung**	0.31 ± 0.25	14.7 ± 12.1	21.8 ± 12.2	15.6 ± 7.71	1.91 ± 1.15	0.00 ± 0.00
**Pancreas**	N.D.	N.D.	0.27 ± 0.12	0.22 ± 0.11	0.15 ± 0.08	N.D.
**Brain**	0.01 ± 0.00	0.01 ± 0.00	0.92 ± 0.48	0.38 ± 0.21	0.64 ± 0.27	0.12 ± 0.06
**Kidneys**	0.07 ± 0.04	0.40 ± 0.20	0.73 ± 0.19	35.5 ± 25.5	0.52 ± 0.14	0.14 ± 0.07
**Liver**	0.37 ± 0.21	71.0 ± 39.8	3.36 ± 0.92	4.34 ± 2.58	2.66 ± 1.37	0.13 ± 0.07
**Spleen**	0.09 ± 0.05	0.04 ± 0.02	5.44 ± 2.04	11.0 ± 6.09	0.53 ± 0.13	0.09 ± 0.05
**Tumor**	0.02 ± 0.01	0.03 ± 0.02	0.20 ± 0.06	0.10 ± 0.05	0.62 ± 0.16	0.13 ± 0.07
**Skin**	0.01 ± 0.01	0.09 ± 0.07	0.11 ± 0.04	0.03 ± 0.01	0.49 ± 0.17	0.09 ± 0.06
**Muscle**	0.02 ± 0.01	0.01 ± 0.01	0.08 ± 0.02	3.23 ± 1.85	0.77 ± 0.30	0.11 ± 0.06

Supplementary Table S2. Parameters in clinical applications of 5-ALA PDT. A summary of currently ongoing 5-ALA PDT clinical trials and reports from clinical trials were used to identify parameters for 5-ALA PDT in this study.

Clinical Trial Code or Citation	5-ALA dose, mg/kg	Drug-light interval	Fluence	Irradiation time	Irradiance	Notes
NCT04391062	-	4-6h	400, 600, 800 J/cm2	-	-	Phase II; Light dose escalation; patients receive maximal safe and intracavitary PDT followed by Stupp protocol radiochemotherapy
NCT04469699	20	3.5-4.5h	-	-	-	Phase II; Stereotactic photodynamic therapy for recurrent GBM with best possible radiochemotherapy at investigator's discretion
NCT03897491	20	3.5-4.5h	-	-	-	Phase II; Safety and efficacy of stereotactic interstitial PDT
Quach *et al* (*3*)	20	3h	mean 12240 J total dose	1h	200 mW/cm	Interstitial PDT on inoperable tumors
Vermandel *et al* (*4*)	20	6h	200 J/cm^2; mean 1772 J total dose	10 mins	-	Post-resection PDT in surgical field

Supplementary Table S3. Summary of studies establishing blood-brain barrier permeabilization parameters. A summary of parameters used in studies utilizing photodynamic priming to permeabilize vessels or to establish safe doses of relative sensitizers. DLI refers to drug-light interval.

Study	Species	Sensitizer Parameters Tested	Light Doses Tested	Permeabilization dose	Damage dose
Semyachkina-Glushkovskaya *et al (5)*	Mouse, brain	5-ALA, 20 and 80 mg/kg, 30 min DLI	10, 15, 20, 40 J/cm^2	20 mg/kg, 15-20 J/cm^2	80 mg/kg
Hirschberg *et al*	Rats, brain	5-ALA, 125 mg/kg, 4h DLI	9 J, 17 J, 26 J	9 J	>17 J
Stummer *et al (6)*	Humans, brain	5-ALA, 0.2, 5, 20 mg/kg, ~3h DLI	--	Goal of fluorescence guided surgery; safe at all doses tested	
Huang *et al (1)*	Mouse, orthotopic pancreatic cancer xenograft	Liposomal VP, 0.25 mg/kg, 1h DLI	100 mW/cm^2, 75 J/cm^2	0.25 mg/kg, 100 mW/cm^2, 75 J/cm^2	--

1. H. C. Huang, S. Mallidi, J. Liu, C. T. Chiang, Z. Mai, R. Goldschmidt, N. Ebrahim-Zadeh, I. Rizvi, T. Hasan, Photodynamic Therapy Synergizes with Irinotecan to Overcome Compensatory Mechanisms and Improve Treatment Outcomes in Pancreatic Cancer. *Cancer Res* **76**, 1066-1077 (2016).
2. M. Radu, J. Chernoff, An in vivo assay to test blood vessel permeability. *J Vis Exp*, e50062-e50062 (2013).
3. S. Quach, C. Schwartz, M. Aumiller, M. Foglar, M. Schmutzer, S. Katzendobler, M. El Fahim, R. Forbrig, K. Bochmann, R. Egensperger, R. Sroka, H. Stepp, A. Rühm, N. Thon, Interstitial photodynamic therapy for newly diagnosed glioblastoma. *Journal of Neuro-oncology* **162**, (2023).
4. M. Vermandel, C. Dupont, F. Lecomte, H.-A. Leroy, C. Tuleasca, S. Mordon, C. G. Hadjipanayis, N. Reyns, Standardized intraoperative 5-ALA photodynamic therapy for newly diagnosed glioblastoma patients: a preliminary analysis of the INDYGO clinical trial. *Journal of Neuro-oncology* **152**, (2021).
5. O. Semyachkina-Glushkovskaya, J. Kurths, E. Borisova, S. Sokolovski, V. Mantareva, I. Angelov, A. Shirokov, N. Navolokin, N. Shushunova, A. Khorovodov, M. Ulanova, M. Sagatova, I. Agranivich, O. Sindeeva, A. Gekalyuk, A. Bodrova, E. Rafailov, Photodynamic opening of blood-brain barrier. *Biomed Opt Express* **8**, 5040-5048 (2017).
6. S. W, S. H, W. OD, P. U, Randomized, Prospective Double-Blinded Study Comparing 3 Different Doses of 5-Aminolevulinic Acid for Fluorescence-Guided Resections of Malignant Gliomas. *Neurosurgery* **81**, 230-239 (2017).
